# Bifurcations of Limit Cycles in a Reduced Model of the *Xenopus* Tadpole Central Pattern Generator

**DOI:** 10.1186/s13408-018-0065-9

**Published:** 2018-07-18

**Authors:** Andrea Ferrario, Robert Merrison-Hort, Stephen R. Soffe, Wen-Chang Li, Roman Borisyuk

**Affiliations:** 1 School of Computing, Electronics and Mathematics, University of Plymouth, Plymouth, UK; 2 School of Biological Sciences, University of Bristol, Bristol, UK; 3 School of Psychology & Neuroscience, University of St Andrews, St Andrews, UK

**Keywords:** *Xenopus* Tadpole, Central Patter Generator, Swimming, Synchrony, Bifurcation Analysis

## Abstract

We present the study of a minimal microcircuit controlling locomotion in two-day-old *Xenopus* tadpoles. During swimming, neurons in the spinal central pattern generator (CPG) generate anti-phase oscillations between left and right half-centres. Experimental recordings show that the same CPG neurons can also generate transient bouts of long-lasting in-phase oscillations between left-right centres. These synchronous episodes are rarely recorded and have no identified behavioural purpose. However, metamorphosing tadpoles require both anti-phase and in-phase oscillations for swimming locomotion. Previous models have shown the ability to generate biologically realistic patterns of synchrony and swimming oscillations in tadpoles, but a mathematical description of how these oscillations appear is still missing. We define a simplified model that incorporates the key operating principles of tadpole locomotion. The model generates the various outputs seen in experimental recordings, including swimming and synchrony. To study the model, we perform detailed one- and two-parameter bifurcation analysis. This reveals the critical boundaries that separate different dynamical regimes and demonstrates the existence of parameter regions of bi-stable swimming and synchrony. We show that swimming is stable in a significantly larger range of parameters, and can be initiated more robustly, than synchrony. Our results can explain the appearance of long-lasting synchrony bouts seen in experiments at the start of a swimming episode.

**Electronic Supplementary Material:**

The online version of this article (10.1186/s13408-018-0065-9) contains supplementary material.

## Introduction

Rhythmic neuronal activity is the basis for many locomotor activities, such as swimming, flying and walking [1–6]. Experimental and modelling evidences suggest that such rhythmicity is generated by specialised neuronal networks called central pattern generators (CPGs) [7, 8]. A key property of a CPG is the ability to autonomously generate rhythmic activity without forcing by periodic external input.

Different motor behaviours require different rhythmic patterns, such as left-right anti-phase oscillations for walking and running [9], or in-phase left-right firing for some forms of crawling [10] and flying [4]. Interestingly, swimming in Xenopus tadpoles follows an anti-phase pattern, but during metamorphosis there is a progressive shift to in-phase limb movements [11]. Although experiments show that some CPG neurons can be active during different motor patterns displaying either in- or anti-phase oscillations [12], it is unclear whether the same group of CPG neurons could be responsible for the generation of these different rhythmic patterns. An alternative hypothesis is that the CPG includes a repertoire of diverse CPG sub-networks, each responsible for a single motor pattern with its own specific firing [13–15].

In this paper, we consider a computational model of the *Xenopus* tadpole CPG. We focus on the neuronal dynamics that drives swimming locomotion in two-day-old tadpoles (two days from fertilisation, developmental stage 37/38). The mechanism for swimming generation is well understood, and previous studies have revealed the detailed structure of the CPG circuit, which is split between left-right sides of the spinal cord (left-right half-centres) spanning the spinal cord and caudal hindbrain [16]. A pattern of swimming-related activity is generated by excitatory descending interneurons (dINs) and inhibitory commissural interneurons (cINs). These key CPG neurons drive the motor response by firing single action potentials per swimming cycle, with firing occurring in anti-phase between left and right half-centres [17].

It has long been known from physiological experiments in tadpoles immobilised with a neuromuscular blocker that the tadpole spinal circuit can also generate a transient form of motor outputs [18] in which there is synchronous firing of neurons between left and right half-centres, with half the swimming period [18–20]. Early simulations suggested that this synchronous output could be stable for neurons excited by positive feedback and coupled by reciprocal inhibition [21, 22]. More recent recordings have confirmed that CPG neurons fire during these transient periods of synchronous activity, which can be spontaneous or induced artificially by injecting constant depolarizing currents [12]. Transitional synchrony may last for a relatively long time (500–1000 ms) and, in most cases, starts shortly after swimming initiation [12, 19]. To date, a behavioural correlate of this pattern has not been characterised, although apparently-pathological “fluttering” movements have been observed (unpublished). It therefore remains unclear whether synchrony is indeed a pathological behaviour or its appearance is an early preparation for a developmental change: during metamorphosis (happening at around 60 days from fertilisation), in-phase and anti-phase motor patterns have been observed/defined both behaviourally and physiologically (ventral root recordings) [11]. We believe that one possible role of synchrony in *Xenopus* tadpoles is to release glutamate/acetylcholine at double the normal swimming frequency in the CPG and at the neuromuscular junctions. This may boost CPG and muscle excitability and help to increase the muscle contraction amplitude/strength at the beginning of swimming.

Our aim is to understand how swimming (anti-phase) and synchrony (in-phase) oscillations can be generated by CPG neurons, find conditions for existence of these two dynamical modes, and for the existence of bi-stability—where both swimming and synchrony can be generated with the same parameters, just by varying the initial stimulus. Furthermore, we seek to understand the mechanism that produces transitions from long-lasting synchrony to stable swimming. In a related work, anti-phase and in-phase oscillations have been found to be stable outputs in recent computational models of the mammalian respiratory CPG [23]. To achieve our goal, we combine a highly reduced neuronal circuit of two pairs of neurons that are known to be essential for the tadpole CPG function [5, 22, 24] with a detailed model of single neuron spiking. Consideration of a small network allows us to use bifurcation analysis for studying the possible dynamical modes. A detailed, biologically plausible model of spike generation allows us to mimic specific features of experimental recordings and compare the results of model simulations with experimental data.

The reduced CPG circuit includes one excitatory (dIN) and one inhibitory (cIN) neuron in each half-centre. Key features of the model include dIN self-excitation acting as a positive feedback and cIN cross inhibition. A circuit with similar characteristics has been studied in [25]. During swimming, this circuit works in the following way. Excitation and subsequent spiking of a dIN leads an ipsilateral cIN to spike, inhibiting the dIN in the opposite half-centre. A key feature of dIN firing is the potential for post-inhibitory rebound (PIR) spiking. Therefore, after some delay the inhibited dIN generates a spike due to PIR, excites the cIN in the same half-centre, and the process repeats to generate an anti-phase spiking pattern between half-centres. During synchrony, dINs on both half-centres fire PIR spikes at similar times, shortly before the arrival of cIN inhibition. When inhibition does arrive, it hyperpolarizes the dINs, which then fire another PIR spike after a relatively slow repolarization period. If synchrony is stable, then the cIN and dIN firing times for the two half-centres become increasingly close together, until both half-centres are firing in perfect synchrony. The activity of dINs drives swimming and other locomotor behaviour by directly exciting the motoneurons that control muscle movement, though we do not include motoneurons in our model [26, 27].

The model of the neuronal circuit includes six synapses, and to model spiking activity, we use a detailed single-compartment Hodgkin–Huxley type model with gating channels’ dynamics motivated by voltage-clamp experiments [28, 29]. Thus, the reduced model includes 34 ordinary differential equations. To study bifurcations, we combine the continuation-based software AUTO-07P [30] and XPPAUT [31]. We study codimension one and two bifurcations of the limit cycles corresponding to swimming and synchrony. This analysis reveals the stability regions for these two limit cycles, including regions where the system can support bi-stable swimming and synchrony. Taking inspiration from the initiation of swimming in real experiments, we formulate a biologically-plausible method of initiating the model’s dynamics based on input currents onto dINs. This initiation procedure allows us to explore to what extent the time jitter and duration between left and right dIN current inputs can lead to stable synchrony or swimming. We show that the swimming mode has a bigger stability region, and it can be initialised for a bigger range of initiation parameter values. This suggests that swimming is the key functional output of young tadpoles. We propose a mechanism for generating long-lasting transient synchrony preceding a swimming episode that is qualitatively similar to synchrony in experimental recordings.

## Methods

### Model Description

The model is a significant reduction of the detailed, biologically realistic model of the swimming network in the tadpole caudal hindbrain and rostral spinal cord, which was described in our previous publications [32–34]. This full model for simulations of the swimming dynamics includes about 2000 neurons and 90,000 synapses with about 200,000 delay differential equations. This model demonstrates a very reliable swimming dynamic under variation of parameter values [34]. In addition, this model has been used to simulate the experimental data of synchrony activity [12].

To use bifurcation analysis for formal mathematical study of the existence and stability of swimming and synchrony, it is necessary to simplify the previous model significantly. Our approach for defining a simplified model for locomotion in tadpoles is to minimise the number of neurons and synaptic connections, and to use a detailed biologically-realistic mathematical description of neurons and synapses. The description of the model comprises two parts.

**Fig. 1:**
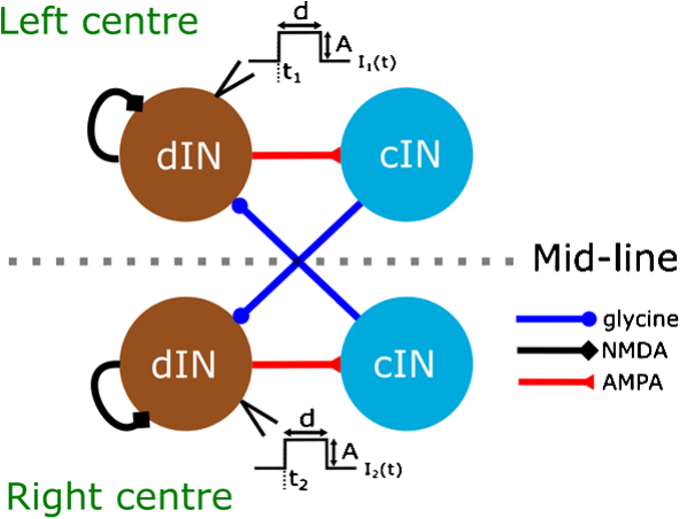
Scheme of neurons and connections in the reduced model. Currents $I_{1} (t)$ and $I_{2} (t)$ represent external depolarizing step currents injected to the two dINs to mimic sensory input. Both currents have the same duration *d* and amplitude *A*. The current pulses for $I_{1} (t)$ and $I_{2} (t)$ are initialised at time $t_{1}$ and $t_{2}$, respectively (see Sect. 3.1 for details)

Firstly, we consider only one “segment” of the spinal cord with the minimal number of neurons in each half-centre needed to characterise the tadpole CPG [5, 24]: one excitatory dIN and one inhibitory cIN. Thus, the “reduced model” includes four neurons, and we assume that the full neuronal network for swimming can be built by expansion of this structure. Figure 1 shows the connections in the reduced model. To compensate for a lack of excitation resulting from removal of synaptic input from other dINs, we introduce dIN self-excitation. In the reduced model we consider identical neurons in both half-centres with symmetrical connections. Therefore, the dynamical system is also symmetrical under mid-line reflection of left and right half-centres (Fig. 1). Secondly, we use a detailed model of spike generation and synaptic transmission to mimic important details of firing patterns in different dynamical modes and compare them with experimental recordings from tadpole neurons. To model neurons’ membrane potential and transmembrane currents, we use the same modified Hodgkin–Huxley spiking model as in the full functional model [34]. To model synaptic connections, we use a similar approach as in the full functional model, the only difference being that in the functional model synapses were modelled using delay differential equations, while here we use synapse models that are continuously dependent on the pre-synaptic potential. We use a continuous model of synaptic transmission because of the difficulties associated with numerical continuation of systems of delay differential equations.

*Neuronal models*. Neuronal spike generation is modelled by the single- compartment Hodgkin–Huxley equations, which includes various types of ionic currents. Although several models describing the activity of dINs and cINs have been developed [17, 22, 24, 34, 35], we believe these models are still not able to reproduce some important properties known from electrophysiology. Here, we use the same neuron models described in [34, 36], because they incorporate some key physiological firing properties detected from experimental recordings [28, 29, 34].

The membrane potential (*v*) of each cell evolves according to equation (1).

1 $$ C \frac{dv}{dt} = i_{\mathrm{lk}} + i_{\mathrm{Na}} + i_{\mathrm{Kf}} + i_{\mathrm{Ks}} + i_{\mathrm{Ca}} + i_{\mathrm{s}} + i_{\mathrm{ext}}, $$

where *C* represents the cell’s capacitance ($C=10$ pF for all neurons). Currents $i_{\mathrm{s}}$ and $i_{\mathrm{ext}}$ represent synaptic and external current sources, respectively. The kinetics of the various ionic channels is based on the previous models of voltage-clamp data [28, 29]. The sodium ($i_{\mathrm{Na}}$), slow potassium ($i_{\mathrm{Ks}}$), fast potassium ($i_{\mathrm{Kf}}$) and leakage ($i_{\mathrm{lk}}$) currents are modelled by traditional Hodgkin–Huxley formalism (equation (2)), while the calcium current ($i_{\mathrm{ca}}$) follows the Goldman–Hodgkin–Katz formulas (equation (3)). For cINs, we set $i_{\mathrm{ca}} =0$.

2 $$\begin{aligned} \begin{aligned} i_{\mathrm{lk}} &= g_{\mathrm{lk}} ( e_{\mathrm{lk}} -v ), \\ i_{\mathrm{Na}} &= g_{\mathrm{Na}} ( e_{\mathrm{Na}} -v ) m^{3} h, \\ i_{\mathrm{Kf}} &= g_{\mathrm{Kf}} ( e_{\mathrm{Kf}} -v ) f^{k}, \\ i_{\mathrm{Ks}} &= g_{\mathrm{Ks}} ( e_{\mathrm{Ks}} -v ) l^{j}, \end{aligned} \end{aligned}$$

3 $$\begin{aligned} i_{\mathrm{ca}} &=2 p_{\mathrm{ca}} {\cdot}\mu{\cdot}F \frac{ [ \mathrm{C a}^{2+} ]_{i} - [ \mathrm{C a}^{2+} ]_{o} \exp ( -\mu )}{1- \exp ( -\mu )} r^{2},\quad \text{where }\mu= \frac{2F{\cdot}v}{ R{\cdot}T}. \end{aligned}$$

Here $g_{\mathrm{lk}}, g_{\mathrm{Na}}, g_{\mathrm{Kf}}, g_{\mathrm{Ks}}$ represent the maximal conductance and $e_{\mathrm{lk}}, e_{\mathrm{Na}}, e_{\mathrm{Kf}}, e_{\mathrm{Ks}}$ represent the equilibrium potential for the leakage, sodium, fast and slow potassium currents, respectively. The values of these parameters are listed in Table 1 for each channel and for both dIN and cIN neurons. All ionic currents depend on one or more voltage-dependent gating variables $m, h, f, l, r$. The constants *k* and *j* represent the powers of the fast and slow potassium gating variables and they are set to values $k=4$, $j=2$ for dINs and $k=1$, $j=1$ for cINs. Parameters of the calcium current are $p_{\mathrm{ca}} =14.25\ \text{cm}^{3}/\text{ms}$, $F=96{,}485~\text{C/mol}$, $R=8.314$ J/(K mol), $T=300$ K, $[ \mathrm{C a}^{2+} ]_{i} = 10^{-7}\ \text{mol/c m}^{3}$, $[ \mathrm{C a}^{2+} ]_{o} = 10^{-5} \ \text{mol/cm}^{3}$.

**Table 1:** Maximal conductance (in nS) and equilibrium potential (in mV) of each ionic channel in the model neurons

	$g_{\mathrm{lk}}$	$e_{\mathrm{lk}}$	$g_{\mathrm{Na}}$	$e_{\mathrm{Na}}$	$g_{\mathrm{Kf}}$	$e_{\mathrm{Kf}}$	$g_{\mathrm{Ks}}$	$e_{\mathrm{Ks}}$
dIN	1.4	−52	240.5	50	12	−80	9.6	−80
cIN	2.47	−61	110	50	8	−80	1	−80

Equation (4) describes the dynamics of each gating variable *x*, $x\in\{m, h, f, l, r\}$, where the voltage-dependent functions $\alpha_{x} (v)$ and $\beta_{x} (v)$ describe the rate of transitions between open and closed states for each ion channel according to (5). The values of the rate parameters A, B, C, D and E for both dINs and cINs are given in the Additional file 1 (Table S1).

4 $$\begin{aligned} \frac{dx}{dt}& = \alpha_{x} ( v ) ( 1-x ) - \beta_{x} ( v ) x, \end{aligned}$$

5 $$\begin{aligned} \alpha_{x} ( v ), \beta_{x} (v)&= \frac{A+Bv}{ C+\exp((D+v)/E)}. \end{aligned}$$

#### Remark

In the case of dINs, the mechanism of PIR is based on de-inactivation of depolarization-activated inward currents [26, 27, 37]. However, the complete mechanism underlying PIR in tadpole dINs still awaits physiological characterization. It is known that, during swimming, dINs are depolarised due to summated, long-lasting NMDA-receptor mediated excitation, and the inhibition leading to PIR occurs against the background of this depolarisation [26].

**Fig. 2:**
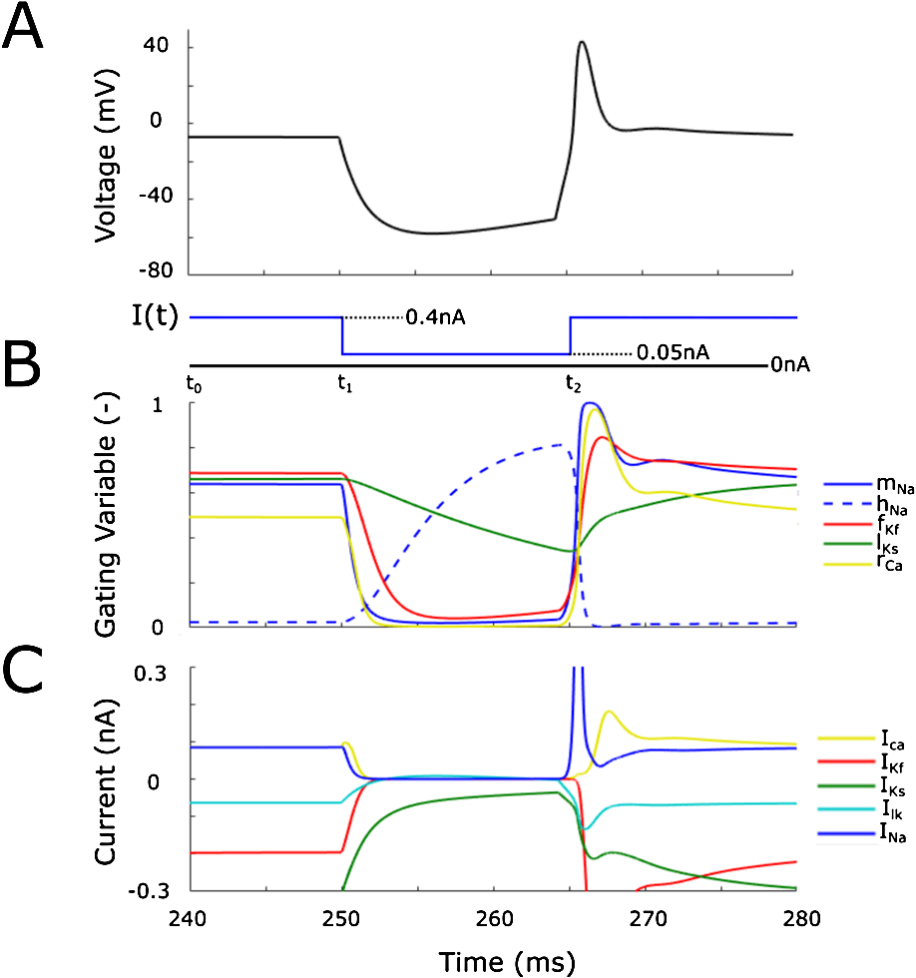
Property of PIR in the dIN model. (**A**) Voltage dynamics in one dIN (black line) during the injection of the current function $I ( t )$ (blue line). (**B**) and (**C**) show the dynamics of the dIN’s gating variables and ionic currents during the same current injection of part (**A**), respectively

Figure 2(A) demonstrates the PIR property of the dIN model. During the time interval $[t_{0}, t_{1}]$, the dIN is in the depolarised state due to constant current injection [34]. During the time interval $[t_{1}, t_{2}]$, the dIN voltage decreases due to the injection of inhibitory current (blue line). Termination of this inhibitory current at time $t_{2}$ (on the background of positive current injection) leads to generation of a dIN spike at time $t_{2}$ via the PIR mechanism.

Figure 2(B)–(C) show the dynamics of the gating variables and ionic currents, respectively. It is clear from these figures that the mechanism of PIR is rather complex due to the interaction of many model components with different time scales. However, we can see how the PIR spike at time $t_{2}$ is triggered by de-inactivation of the sodium current.

*Synaptic models*. The reduced model includes excitatory and inhibitory connections. We consider both AMPA and NMDA receptors of glutamate excitatory synapses from dINs, and glycinergic receptor for inhibitory synapses from cINs (Fig. 1). Summation of the slow synaptic transmission mediated by NMDA receptors from dIN to dIN synaptic transmission is essential for the generation of swimming activity because PIR spiking in dINs needs inhibition to arrive against a sufficiently high level of depolarization [22]. For this reason, we consider NMDA-driven self-excitatory connections in dINs. As in the full model of the swimming network, dINs in the reduced model are able to fire PIR spikes on release from cIN inhibition. The six synaptic connections of the reduced model (Fig. 1) encompass the key properties of the tadpole CPG: ipsilateral excitation (driven by NMDA/AMPA synapse), commissural inhibition (driven by glycinergic synapses) and post-inhibitory rebound in dINs.

Equations (6)–(10) describe the synaptic currents $i_{\mathrm{s}}$ ($\mathrm{s}\in\{\mathrm{ampa},\mathrm{nmda},\mathrm{inh}\}$). The time evolution of every synaptic transmission event depends on the opening and closing of state variables, $o_{\mathrm{s}}$ and $c_{\mathrm{s}}$, respectively. Equation (7) describes the dynamics of these variables: $y_{\mathrm{s}} ( t ),\ y_{\mathrm{s}} \in\{ c_{\mathrm{s}}, o_{\mathrm{s}} \}$. In this equation, we use a well-known model of synaptic transmission that depends continuously on the pre-synaptic membrane potential ${v}_{\mathrm{pre}}$ [38] with $g_{\mathrm{s}} = g_{\mathrm{s}} ( v_{\mathrm{pre}} )$ representing the concentration of released neurotransmitter formulated in (8). In the case of NMDA receptors, voltage-dependence of the synaptic current is described by the factor $\mathrm{Mg}({v})$ representing $\mathrm{Mg}^{2+}$ modulation of NMDA receptors (equations (9)–(10)).

6 $$\begin{aligned} i_{\mathrm{s}}& = w_{\mathrm{s}} ( e_{\mathrm{s}} -v ) ( c_{\mathrm{s}} - o_{\mathrm{s}} ), \end{aligned}$$

7 $$\begin{aligned} \frac{d y_{\mathrm{s}}}{dt}& = g_{\mathrm{s}} ( v_{\mathrm{pre}} ) ( 1- y_{\mathrm{s}} ) - \frac{y_{\mathrm{s}}}{\tau_{y}^{\mathrm{s}}}, \end{aligned}$$

8 $$\begin{aligned} g_{\mathrm{s}} ( v_{\mathrm{pre}} )&= \frac{T_{\mathrm{s}}}{1+\exp ( \overline{v}_{\mathrm{s}} - v_{\mathrm{pre}} )}, \end{aligned}$$

9 $$\begin{aligned} i_{\mathrm{nmda}}& = w_{\mathrm{nmda}} ( e_{\mathrm{nmda}} -v ) ( c_{\mathrm{nmda}} - o_{\mathrm{nmda}} ) {\cdot}\mathrm{Mg} ( v ), \end{aligned}$$

10 $$\begin{aligned} \mathrm{Mg} ( v ) &=1/(1+0.05 \exp ( -0.08v ), \end{aligned}$$

Here the values of parameters $e_{\mathrm{s}}, \tau_{o}^{s} \tau_{c}^{s},\overline{v}_{s} $ and $T_{\mathrm{s}} $ are given in Table 2. Parameters $w_{\mathrm{s}}$ and $e_{\mathrm{s}} $ represent the synaptic strength and reversal potential of each type of synapse, respectively. The time constants $\tau_{o},\tau_{c}$ of the opening and closing state variables *o* and *c* have been fitted from pairwise electrophysiological recordings [39] and follow the time course of the different receptor types. The slow de-inactivation of the NMDA is important for a proper functioning of swimming [39]. We do not investigate the variation of these time constants. Parameters $w_{\mathrm{ampa}}$, $w_{\mathrm{nmda}}$ and $w_{\mathrm{inh}}$ are the bifurcation parameters that we varied during numerical continuation. In the results section we will discuss the values of these parameters.

**Table 2:** Parameters of the synaptic models

s	NMDA	AMPA	glycine
${e}_{\mathrm{s}}$ (mV)	0	0	−75
$\tau_{\mathrm{o}}^{\mathrm{s}}$ (ms)	0.5	0.2	1.5
$\tau_{\mathrm{c}}^{\mathrm{s}}$ (ms)	80	3.0	4.0
$\overline{{v}}_{\mathrm{s}}$	10	10	1
${T}_{\mathrm{s}}$	1.5	4.0	1

#### Remark

Parameter $w_{\mathrm{ampa}}$ describes the connection strength of the dIN→cIN coupling (for simplicity, we consider the dynamics of the AMPA synapse only). We calculate the physiological range of variation for this parameter using the following experimental findings: (1) The dINs spike reliably and synchronously during each swimming cycle [12, 27]; (2) The average number of incoming connections from dINs to cINs participating in swimming is in the range (15, 17) [36]; (3) The maximal unitary strength of the AMPA synapse is 0.6 nS [39]. Thus, it gives the physiological range of parameter $w_{\mathrm{ampa}}$: (9 nS, 10.2 nS).

Parameter $w_{\mathrm{nmda}}$ describes the connection strength of the dIN→dIN coupling. For simplicity, we consider the dynamics of a slow NMDA synapse only, but adjust the connection strength to reflect the fast AMPA component as well. To calculate the physiological range of this parameter variation, we use experimental findings similar to the consideration above. The average number of incoming connections to dINs from dINs is in the range (13–21) [36], and the maximal unitary strengths of the AMPA and NMDA synapses are 0.6 nS and 0.15 nS, respectively [39]. To take into account the AMPA influence, we adjust the strength by summing these values and multiply by the range of incoming connections to get the physiological range of parameter $w_{\mathrm{nmda}}$: (10 nS, 15.8 nS).

For our numerical study of bifurcations, we widen the range for both $w_{\mathrm{ampa}}$ and $w_{\mathrm{nmda}}$ to clarify the relationship between different bifurcations (e.g. to find the turning point). Therefore, we vary the parameters $w_{\mathrm{ampa}}$ and $w_{\mathrm{nmda}}$ in the ranges (9 nS, 20 nS) and (8 nS, 20 nS), respectively.

### Software

For numerical studies of limit cycles, we combine several software tools. To run numerical integration and find periodic orbits, we use XPPAUT [31] with the CVODE variable time step integrator with absolute and relative tolerances equal to 1e–12. We use the stable periodic orbit to start numerical continuation in order to determine stability and find bifurcations. To perform numerical continuation and detect the bifurcations of the reduced model, we use the software package AUTO-07P [30]. We use custom written Python code to transform equations, variables, functions and parameters from XPPAUT to AUTO. To study the initiation of the stable limit cycles and run multiple numerical integrations in parallel, we use both XPPAUT and custom written MATLAB code (MathWorks, Inc) with different variable time step integration schemes (ode23tb, ode45) to confirm the accuracy of our results. To integrate the system with noise, we use standard Euler–Maruyama method with time step $dt=0.01$.

## Results

### Swimming and Synchrony Limit Cycles

In this section, we validate the reduced model by showing that it can produce activity similar to that seen in experimental recordings. To do so, we fix synaptic strengths and simulate the reduced model to reproduce swimming and synchrony dynamics.

In experiments with immobilised tadpoles, CPG neurons are normally at rest before the start of a swimming episode. This start is marked by a gradual depolarization of the membrane potential that can lead to rhythmic firing [40]. To mimic these experiments, we initialise neurons at rest, and we use the following initiation procedure to control perturbations and move the orbit from the resting state to a basin of attraction of either swimming or synchrony.

*Initiation of the dynamics*. In experiments, a swimming episode can start after brief head or trunk skin stimulation on one side of the animal [41, 42]. Skin stimulation leads to neuronal firing in the sensory pathway, which delivers, with some delay, excitation to CPG neurons in both half-centres. Experiments have shown that the start of movement occurs shortly after the first dINs spikes [41], and that dIN activity drives spiking of other neurons during swimming [27].

To move the system out of its initial rest state and initiate activity in the reduced model, we inject a depolarizing step current $i_{\mathrm{ext}}$ with fixed amplitude $A=0.1$ (nA) and duration *d* (ms) to dINs in the left and right half-centres at times $t_{1}$ and $t_{2}$, respectively, where time delay $\Delta= t_{2} - t_{1}$ (Fig. 1).

**Fig. 3:**
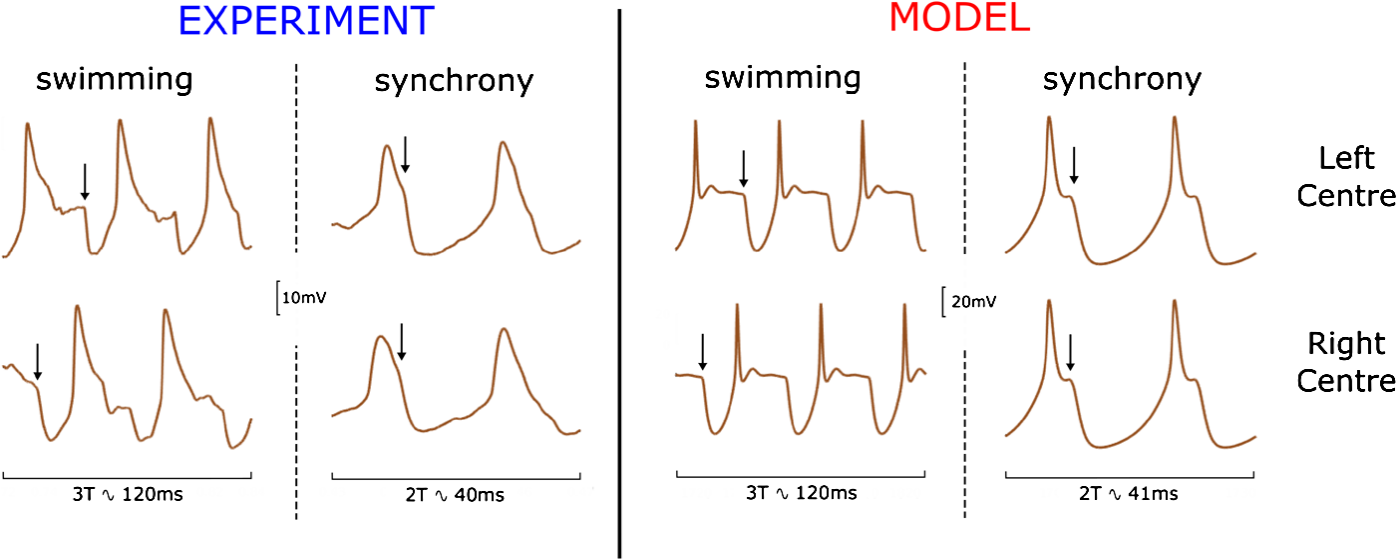
Voltage traces of dINs in the reduced model during swimming and synchrony. The left panel shows experimental pairwise recordings from one left-centre dIN and one right-centre dIN during swimming and synchrony. The right panel shows the voltage variable of the two dINs of the reduced model during swimming and synchrony. We show three cycles of swimming (3T) and two cycles of synchrony (2T) to highlight the characteristic shapes of the membrane potential during these two regimes and to compare experiments and model simulations. Arrows indicate the firing of cINs and mark the inhibition preceding PIR spikes in dINs. Model parameters used to obtain swimming are ${w}_{\mathrm{inh}} =23$ nS, ${w}_{\mathrm{ampa}} =12$ nS and ${w}_{\mathrm{nmda}} =10$ nS, with initiation parameters $\Delta=140$ ms and ${d}=6$ ms. Model parameters used to obtain synchrony are ${w}_{\mathrm{inh}} =55$ nS, ${w}_{\mathrm{ampa}} =12$ nS and ${w}_{\mathrm{nmda}} =10$ nS, and initiation parameters $\Delta=0$ ms and $\mathrm{d}=6$ ms. The experimental recordings have been obtained using the same experimental protocols and conditions described in [12]

We use the initiation procedure to run numerical integration of the reduced model in order to find stable oscillatory regimes. Figure 3 shows both experimental recordings (left panel) and stable regimes of the reduced model (right panel). The left part of each panel shows the membrane potential of dINs in each half-centre of the body during swimming, and the right part shows the membrane potentials during synchrony. Parameter values for these simulations are given in the figure caption.

Although the model describes a highly reduced CPG, the pattern of dIN membrane potential trajectories qualitatively matches the experimental recordings well. These typical spiking patterns of swimming and synchrony modes include dIN post-spike depolarization and deep inhibition (black arrows show time of cIN spikes in the opposite half-centres) causing inhibition and subsequent rebound spiking. These two typical oscillatory patterns correspond to limit cycles in the phase space of the dynamical system. The swimming mode with anti-phase oscillations in opposite half-centres corresponds to the Swimming limit Cycle (SwC), while the synchrony mode of in-phase oscillations corresponds to Synchrony limit Cycle (SyC).

### Symmetry in the Reduced Model

From Fig. 1, one can see that the reduced model is invariant under reflection of neurons and synapses on the mid-line. This means that the reduced model is a $\mathbb{Z}_{2}$-equivariant dynamical system. The reduced model can be written in the general form of an *n*-dimensional system, where $n=2k$ and *k* is the number of equations describing the dynamics of the variables related to the left and right half-centres:

11 $$ \dot{y} =f ( y ),\quad y\in \mathbb{R}^{2k}. $$

We arrange the equations in such a way that the first *k* equations describe the state variables of neurons and synapses in the left half-centre as well as the commissural synaptic connection from left cIN to right dIN. We denote all variables related to the left half-centre by vector $y_{L} (t),\ y_{L} \in \mathbb{R}^{k}$. The other *k* equations likewise describe neuronal variables and synaptic connections in the right half-centre, as well as the commissural synaptic connection from right cIN to left dIN. We denote these right half-centre variables by vector $y_{R} (t),\ y_{R} \in \mathbb{R}^{k}$. The system is symmetrical because the equations for variables of the left and right half-centres in (1) are identical. If we swap variables $y_{L}$ and $y_{R}$ in (11), then the equations for $y_{L}$ become equations for $y_{R}$ and these equations are equivalent to the equations for $y_{R}$ in (11). An equivalent statement is valid for the $y_{R}$ equations.

It follows from the system’s symmetry that any limit cycle that exists in system (11) is of one of three types:

Type (1) **In-phase** limit cycle: $y_{L} ( t ) = y_{R} ( t ),\ \forall t$.
Type (2) **Anti-phase** limit cycle: $y_{L} ( t ) = y_{R} ( t+T/2 ),\ \forall t$, here *T* is period of oscillation.
Type (3) **Out-of-phase** limit cycle: $y_{L} ( t ) = y_{R} ( t+P ),\ \forall t$, here $P\neq T/2$ is phase shift.
Type (4) **Asymmetrical** limit cycle: $y_{L} ( t ) \neq y_{R} ( t+P ),\ \forall t$, ∀*P*.

It is clear that the synchrony limit cycle SyC should be of type (1), and this cycle belongs to the symmetry manifold $Y_{k}^{+} = \{ y\in \mathbb{R}^{2k}: y_{L} = y_{R} \}$. The swimming limit cycle SwC should be of type (2). All limit cycles of type (3–4) should exist in pairs.

*Initiation with symmetry*. By selecting proper values for the initiation parameters described in Sect. 3.1, we can initiate limit cycles of different types. For example, to initiate the dynamics inside the in-phase manifold $Y_{k}^{+}$, we select $\Delta =0$. This means that dINs in both half-centres simultaneously receive the same stimulating input; therefore, the orbit is locked inside the manifold $Y_{k}^{+}$. If $\Delta\neq0$, the dynamics are initialised outside the manifold $Y_{k}^{+}$, and an orbit can be either attracted to a stable attractor inside of the manifold $Y_{k}^{+}$ or repulsed from the manifold.

### Bifurcation Analysis Under One Parameter Variation

In this section we use bifurcation theory to study dynamical regimes in the reduced model under variation of one parameter. We begin from motivation of the choice of bifurcation parameters used for both codimension-one and codimension-two studies.

*Choice of the bifurcation parameters*. We assume that the values of all model parameters are fixed except for three parameters which we vary in turn using numerical continuation. All parameter values governing the intrinsic dynamics of the neurons are selected according to our previous study of the full physiological model [34]. Many of these parameter values have been directly measured in experiments, although some were selected from a physiological range in model simulations. Values of these neuronal parameters are fixed for the purposes of bifurcation analysis. The three parameters that we vary, $w_{\mathrm{ampa}}$, $w_{\mathrm{nmda}}$ and $w_{\mathrm{inh}}$, correspond to synaptic strengths for excitatory and inhibitory synapses.

We choose to vary these parameters for three reasons. Firstly, although these parameters are important for reliable functioning of the CPG and, in particular, for reliable swimming, it is difficult to measure their values in experiments. Simulations of the full physiological model show that the swimming regime is very robust: swimming exists even when these parameter values are varied in a wide range [34]. However, in a recent work [43] we investigated the effect of axon fasciculation in the spinal network, and we found that a proper balance between excitatory and inhibitory connection strengths is needed for generating a reliable CPG swimming activity. Secondly, experimental recordings [12] show that occasional synchrony appears more frequently soon after a stimulus that initiates swimming, at a time when excitatory drive is stronger than during later swimming [44]. Moreover, synchrony appears less frequently when glycinergic inhibition is artificially reduced by application of inhibitory blockers [12]. We hypothesise that these excitatory and inhibitory contributions are mainly driven from cINs and dINs. Thirdly, a previous experimental work [45] showed how strong background excitation and phasic inhibition can influence the swimming period. We used the reduced model to explore how variations in excitatory and inhibitory strengths shape the period of the synchrony and swimming limit cycles. The strength of the conductance driven by dINs and cINs synaptic transmissions represents two major contributions of these two components. By computing the period of synchrony and swimming limit cycles under variation of the synaptic strengths, we explored changes in the swimming and synchrony periods.

By selecting these parameters for bifurcation analysis, we aim to find the critical boundaries of stability for the swimming and synchrony modes. Since swimming is the main functional behaviour of the animal at the considered stage of development, we expect that its stability region would most likely occupy a large area in parameter space. Therefore, we first study bifurcations under variation of inhibitory connection strength $w_{\mathrm{inh}}$. We then study codimension-two bifurcations by varying $w_{\mathrm{inh}}$ together with either $w_{\mathrm{ampa}} $ or $w_{\mathrm{nmda}}$ (Sect. 3.4). Throughout the following sections we use the same notation when referring to codimension-one bifurcation points in two-dimensional space and to their horizontal coordinate.

**Fig. 4:**
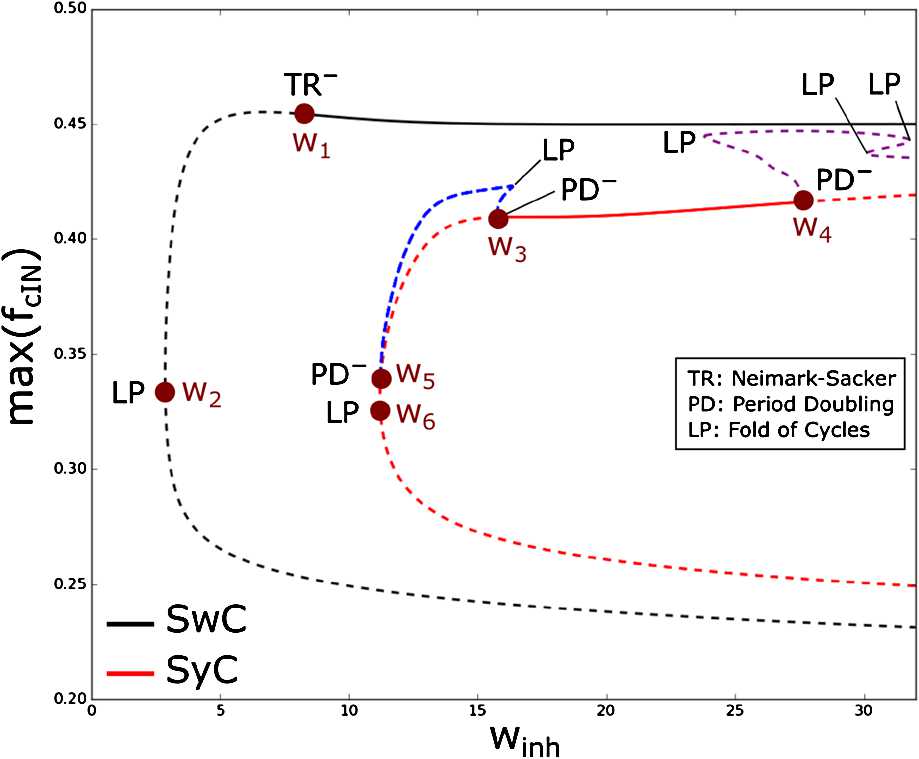
One-dimensional bifurcation diagram for the swimming (black) and synchrony (red) limit cycles at varying inhibitory strength $w_{\mathrm{ihn}}$. Blue and purple lines show two unstable limit cycles appearing at bifurcation points $w_{3}$ and $w_{4}$, respectively. The y-axis shows the maximum of the $K_{f}$-gating variable *f* of the left cIN for each limit cycle. Stable and unstable limit cycles are shown by continuous and dashed lines, respectively. The superscript − refers to subcritical bifurcations. Bifurcation parameter values (in nS) are the following: $w_{1} =8.57$, $w_{2} =2.86$, $w_{3} =15.74$, $w_{4} =27.6$, $w_{5} =11.23$ and $w_{6} =11.21$

We begin with the study of bifurcations of the swimming and synchrony limit cycles under variation of the inhibitory strength $w_{\mathrm{inh}}$. We use each stable limit cycle as a starting point for a numerical continuation procedure. In Fig. 4 we show continuation of the SwC (black curve) and SyC (red curve) under variation of parameter $w_{\mathrm{inh}}$, and we fix parameter values $w_{\mathrm{ampa}} =12$ nS and $w_{\mathrm{nmda}} =10$ nS.

In Fig. 4 the black curve shows that the SwC is stable for $w_{\mathrm{inh}} > w_{1}$. The critical parameter value $w_{\mathrm{inh}} = w_{1}$ corresponds to a subcritical Neimark–Sacker (torus) bifurcation (TR^−^). At this critical parameter value, the stable SwC becomes unstable for $w_{\mathrm{inh}} < w_{1}$ merging with an unstable torus (torus continuation is not shown in Fig. 4) which co-exists with the stable SwC for $w_{\mathrm{inh}} > w_{1}$. Thus, the SwC is unstable (dashed black line) for $w_{\mathrm{inh}} < w_{1}$. At the critical parameter value $w_{\mathrm{inh}} = w_{2},\ (w_{2} < w_{1} )$ this unstable SwC cycle disappears via a fold (limit point) bifurcation (LP) by merging with another unstable cycle.

#### Remark

Our calculations show that stable SwC can be continued until very large values of $w_{\mathrm{inh}} \sim1000$ (nS) (not shown).

In Fig. 4, the solid red line corresponds to the stable SyC for $w_{\mathrm{inh}} \in( w_{3}, w_{4} )$. Both critical parameter values $w_{\mathrm{inh}} = w_{3}$ and $w_{\mathrm{inh}} = w_{4} $ correspond to subcritical period-doubling bifurcations (PD^−^). At a critical parameter value $w_{\mathrm{inh}} = w_{3}$ the stable SyC merges with the unstable limit cycle of double period (blue dashed line) which exists for $w_{\mathrm{inh}} > w_{3}$ and becomes unstable for $w_{\mathrm{inh}} < w_{3}$. Similarly, at the critical parameter value $w_{\mathrm{inh}} = w_{4}$ the stable SyC merges with the unstable limit cycle of double period (purple dashed line) which exists for $w_{\mathrm{inh}} < w_{4}$ and becomes unstable for $w_{\mathrm{inh}} > w_{4}$. The dashed red line shows the unstable SyC.

It is interesting to note that detailed study of these two unstable limit cycles of double period reveals that these cycles are of two different types (blue and purple lines). The limit cycle shown by the blue line is of type (1), and it belongs to the symmetry manifold $Y_{k}^{+}$. Further investigation of this blue cycle reveals a fold bifurcation and another subcritical period-doubling bifurcation ($w_{\mathrm{inh}} = w_{5} $). As a result of this subcritical period-doubling bifurcation, the unstable limit cycle of double period (blue dashed line) merges with the unstable SyC (red dashed line) inside of the symmetry manifold $Y_{k}^{+}$. The unstable SyC disappears via a fold bifurcation ($w_{\mathrm{inh}} = w_{6} $).

The limit cycle shown by the purple line is of type (2), and this cycle lies outside the symmetry manifold $Y_{k}^{+}$. Further bifurcations of this unstable limit cycle of double period include several fold bifurcations where two unstable limit cycles merge and disappear.

This analysis shows that there is a region of bi-stability $w_{3} < w_{\mathrm{inh}} < w_{4}$ for the SwC and the SyC limit cycles. We notice that the range of parameter values where the SwC is stable is significantly larger than that of the range where the synchrony cycle is stable.

### Stability of Swimming and Synchrony Under Variation of Two Parameters

In this section we consider bifurcations of swimming and synchrony cycles under two-parameter variation. We vary the synaptic strength of inhibition $w_{\mathrm{inh}}$ with either $w_{\mathrm{nmda}}$ or with $w_{\mathrm{ampa}}$.

**Fig. 5:**
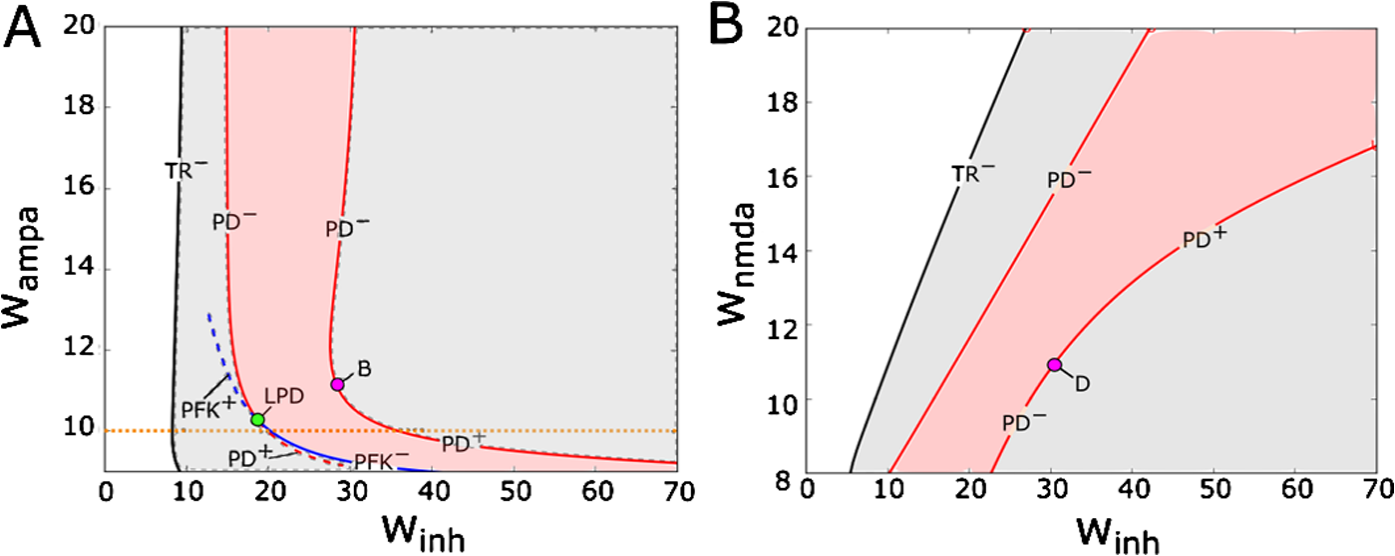
Codimension-two bifurcation diagrams showing the stability regions for the swimming (light grey and red) and synchrony (light red) limit cycles under variation of ($w_{\mathrm{inh}}$, $w_{\mathrm{ampa}}$) in (**A**) and ($w_{\mathrm{inh}}$, $w_{\mathrm{nmda}}$) in (**B**). Superscripts − and + refer to subcritical and supercritical bifurcations, respectively. To clarify the stability of the limit cycles for low values of $w_{\mathrm{ampa}}$, we computed the codimension-one bifurcation diagram at fixed value $w_{\mathrm{ampa}} =10$ nS shown in Fig. 6 (orange dotted line). Bifurcation points *B* and *D* switch the criticality of the PD bifurcation (subcritical to supercritical). The LPD point is a fold-flip bifurcation point. At this point, a pitchfork bifurcation curve (PFK, blue line) interacts with a PD line and both exchange criticality

Figure 5 shows the two-dimensional stability regions of swimming and synchrony cycles under variation of parameter pairs ($w_{\mathrm{inh}}, w_{\mathrm{ampa}} $) (Fig. 5(A)) and ($w_{\mathrm{inh}}, w_{\mathrm{nmda}} $) (Fig. 5(B)). In both figures, the grey area shows the stability region of the swimming limit cycle, and inside this area is a light red shaded area corresponding to stability of the synchrony cycle. In fact, this light red area shows the region of bi-stability, where both SwC and SyC are stable. The white area in the left part of each panel corresponds to the stationary state without oscillations. From the figures it is clear that the synchrony cycle has a smaller stability region regardless of which excitatory synaptic strength is changed.

In both Fig. 5(A) and (B), the critical boundary (black line marked by TR^−^) of the SwC stability region corresponds to a subcritical Neimark–Sacker (torus) bifurcation. On the right of this line, a stable SwC co-exists with an unstable torus. The SwC and the torus merge and disappear on the critical boundary.

The stability region of the SyC is limited by two period-doubling bifurcation lines (red). In Fig. 5(A), both critical boundaries correspond to sub-critical period-doubling bifurcations for larger values of $w_{\mathrm{ampa}}$ (red lines marked PD^−^). For smaller values of $w_{\mathrm{ampa}}$ both period-doubling boundaries become supercritical (red line marked PD^+^). We note that everywhere on the period-doubling bifurcation line (red) one multiplier is (−1).

**Fig. 6:**
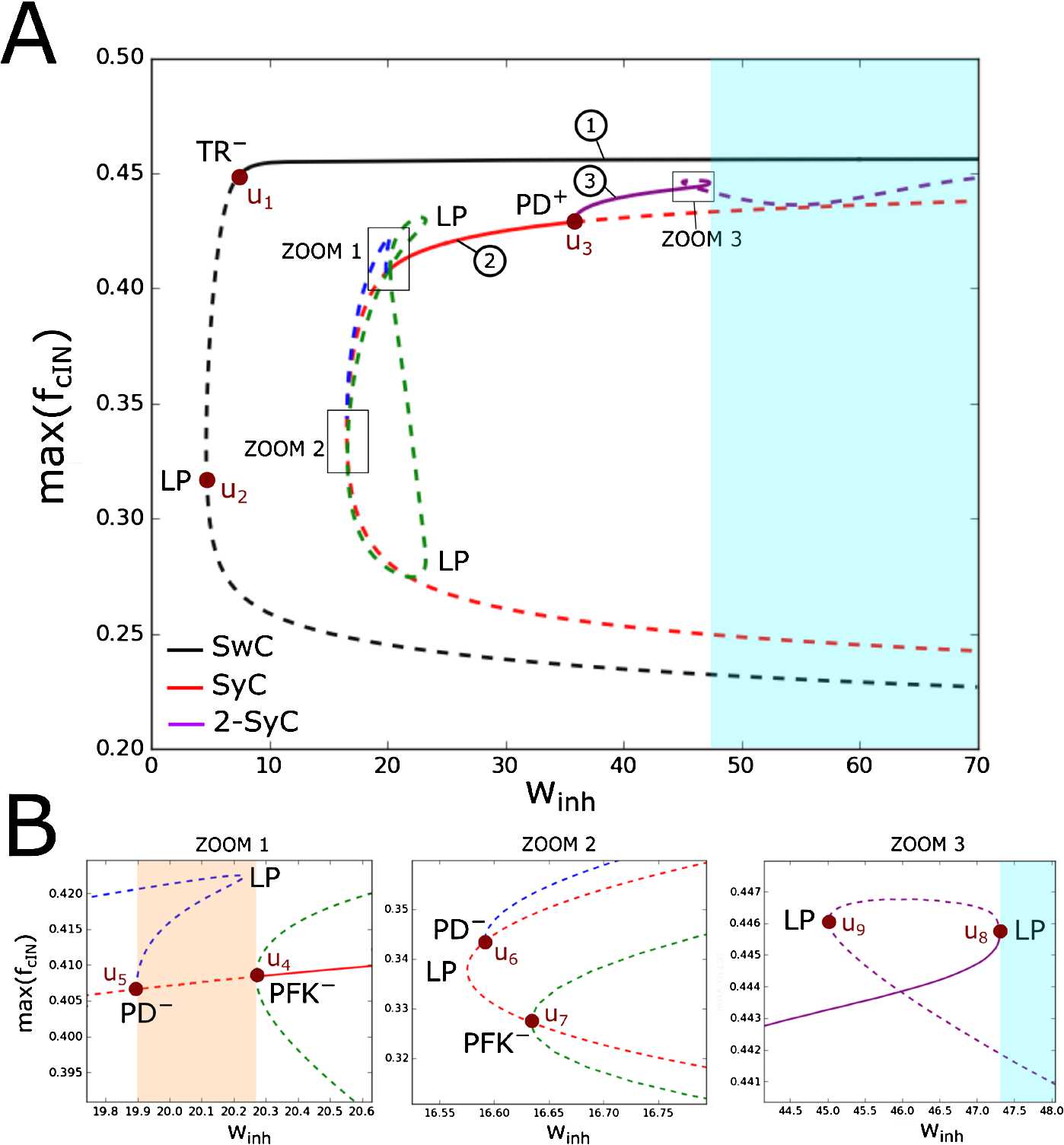
(**A**) One-dimensional bifurcation diagram for the synchrony (red), swimming (black) and double-synchrony (purple) limit cycles at varying inhibitory strength $w_{\mathrm{ihn}}$ and fixed parameters $w_{\mathrm{ampa}} =10$ nS and $w_{\mathrm{nmda}} =10$ nS. The y-axis shows the maximum of the $K_{f}$-gating variable *f* of the left cIN for each limit cycle. Blue and green lines show unstable limit cycles appearing at bifurcation points $u_{5}$ and $u_{4}$, respectively. Stable and unstable limit cycles are shown by continuous and dashed lines, respectively. The superscript − refers to subcritical bifurcations. (**B**) Zoom of selected regions of Fig. 6(A)

On the left critical boundary there is a point corresponding to a codimension two fold-flip bifurcation (green point marked LPD). At this bifurcation point, one additional multiplier becomes equal to the critical value (+1). It is known from [46] that the bifurcation diagram in the vicinity of LPD critical point is very complex, and there are several bifurcation lines, which intersect at such bifurcation point. [46] shows the bifurcation diagram near the LPD point. It is clear from this diagram that at this bifurcation point the period-doubling line changes from sub- to supercritical. In addition, the diagram shows that the period-doubling line and the fold bifurcation line intersect at the LPD point. Possibly there are other bifurcation lines interacting in a LPD bifurcation, which we did not find. Since our model is symmetrical, it is possible that our system has a pitchfork line instead of a fold line, and that this pitchfork line interacts with a period-doubling line in a symmetrical version of the LPD bifurcation. To clarify the boundary of SyC stability near the LPD point, we fix the parameter value $w_{\mathrm{ampa}} =10 $ and vary only one parameter $w_{\mathrm{inh}}$ to find bifurcations (horizontal dotted orange line in Fig. 5(A)). Figure 6 shows the results of this analysis. In particular, the panel ZOOM 1 of Fig. 6(B) shows that there are two bifurcations in the area of interest. The critical parameter value $w_{\mathrm{inh}} = u_{4}$ corresponds to the subcritical pitchfork of limit cycles bifurcation (red dot $u_{4}$ marked PFK). The SyC is stable in region $w_{\mathrm{inh}} > u_{4}$, and it becomes unstable for $w_{\mathrm{inh}} < u_{4}$. At the PFK^−^ parameter $w_{\mathrm{inh}} = u_{4}$ a pair of unstable out-of-phase limit cycles of type (4) merge and disappear (green lines in Fig. 6). This has an important implication used in Sect. 3.7: When the stable SyC becomes unstable at critical point $u_{4}$, the loss of stability is in the transversal direction to the symmetry manifold $Y_{k}^{+}$. In addition, the panel ZOOM 1 in Fig. 6(B) shows the period-doubling bifurcation of unstable SyC ($w_{\mathrm{inh}}^{\mathrm{cr}} = u_{5}$).

We use the critical parameter value of subcritical pitchfork bifurcation $w_{\mathrm{inh}}^{\mathrm{cr}} = u_{4}$ to start a new continuation under variation of two parameters, and the result is shown in Fig. 5(A) by a solid blue line marked PFK^−^. The intersection of this line with the stability region causes the stable SyC to become unstable via subcritical pitchfork bifurcation.

#### Remark

There are several unstable limit cycles in Fig. 6 shown by dashed green lines (type (4) out-of-phase cycles) and blue lines (type (2) limit cycle of double period). The critical parameter values $w_{\mathrm{inh}}^{\mathrm{cr}} = u_{5}$ and $w_{\mathrm{inh}}^{\mathrm{cr}} = u_{6}$ correspond to period-doubling bifurcations and $w_{\mathrm{inh}}^{\mathrm{cr}} = u_{7}$ corresponds to the pitchfork bifurcation.

Now we return to the SyC stability region in Fig. 5(A) and consider the right boundary (red line) which corresponds to the period-doubling bifurcation.

If $w_{\mathrm{ampa}} =12$, then we know from Fig. 4 that the period-doubling bifurcation at $w_{\mathrm{inh}}^{\mathrm{cr}} = w_{4}$ is subcritical. If $w_{\mathrm{ampa}} =10$, then we know from Fig. 6(A) that the period-doubling bifurcation at $w_{\mathrm{inh}}^{\mathrm{cr}} = u_{3}$ is supercritical: the stable SyC becomes unstable and a stable limit cycle of double period appears. Stable double-period cycle is an in-phase type (1) limit cycle. Here we introduce the notation 2-SyC for this synchrony cycle of double period. Some additional details of the evolution of this limit cycle (solid purple line in Fig. 6(A)) are shown in Fig. 6(B), panel ZOOM 3. This means that somewhere between these two points of the period-doubling bifurcation line (($w_{4}$, 12) and ($u_{3},\ 10$)) should be some bifurcation point (*B*), which corresponds to this change. At this point (*B*) the red line of subcritical period doubling (marked PD^−^) becomes the line of supercritical period-doubling bifurcation (marked PD^+^ at Fig. 5(A)). We are unable to find point *B* via computational continuation. Therefore, to calculate the coordinates of this point, we use multiple simulations of the reduced model to find where the double-period limit cycle is stable outside of the SyC stability region. We started simulations from the following point ($\Delta =0.1$, $d=6$, $w_{\mathrm{ampa}} =10$ nS, $w_{\mathrm{nmda}} =10$ nS, $w_{\mathrm{inh}} =42$ nS) and slightly varied parameters ($w_{\mathrm{inh}}$, $w_{\mathrm{ampa}}$), decreasing the value of $w_{\mathrm{ampa}}$ to find the double-period cycle and define its stability. As a result, we find the coordinates of point *B* on the period-doubling line: (27.8, 11.5).

**Fig. 7:**
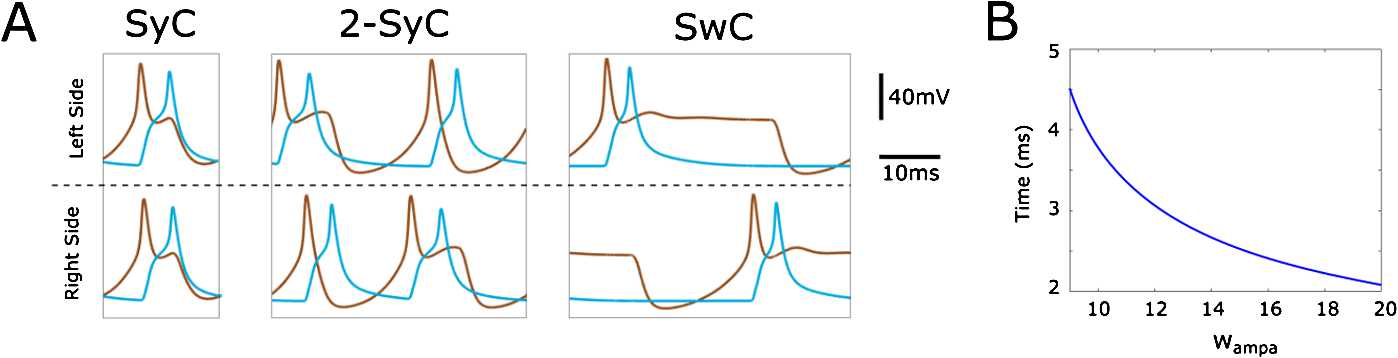
(**A**) Time evolution of the voltage of dINs (brown lines) and cINs (blue lines) during one period of synchrony (SyC), double-synchrony (2-SyC) and swimming (SwC). The three limit cycles are detected by AUTO in Fig. 6. Synaptic strength parameters used to generate each panel SyC, 2-SyC and SwC, respectively, are $w_{\mathrm{inh}} =30$ nS, 45 nS and 40 nS. (**B**) Time difference between left dIN and left cIN spikes during one cycle of the SwC at varying $w_{\mathrm{ampa}}$. The remaining vector of parameters used to obtain this figure are $w_{\mathrm{inh}} =60$ nS, $w_{\mathrm{nmda}} =10$ nS, $\Delta=50$ ms and $d=6$ ms

Figure 7(A) shows the voltage traces of the model neurons for each of the three stable limit cycles (SwC, SyC and 2-SyC). Each neuron fires once per cycle in the cases of SyC and SwC, and it fires twice per cycle in the case of 2-SyC. For each limit cycle, dIN firing evokes a single spike in the ipsilateral cIN. Clearly, the timing of cIN firing depends on the strength of the AMPA synapses $w_{\mathrm{ampa}}$.

In Fig. 7(B) we show the time difference between left cIN and left dIN spikes during swimming as a function of $w_{\mathrm{ampa}}$ (for fixed parameters $w_{\mathrm{nmda}} =10$ nS and $w_{\mathrm{inh}} =40$ nS).

Now we consider Fig. 5(B), which shows the stability region of the SyC cycle under variation of ($w_{\mathrm{inh}}, w_{\mathrm{ndma}} $). This region is shown by red shading, and the two boundaries (left and right red lines) correspond to period-doubling bifurcations. Using simulations of the reduced model, we find that the left line corresponds to the subcritical period-doubling bifurcation (marked PD^−^).

Analysing the right boundary, we find that this period-doubling bifurcation line is supercritical for high values of $w_{\mathrm{nmda}}$ and it becomes subcritical for low values of $w_{\mathrm{ndma}}$ at some bifurcation point *D* represented in Fig. 5(B). Point *D* was not detected by AUTO, so to find its coordinates we used simulations in a similar way as described above for finding the coordinates of point (*B*) in Fig. 5(A). As a result, we find the coordinates of point *D* on the period-doubling line: (32.6,11.4).

#### Remark

As we have seen above, the bifurcation software AUTO cannot reliably distinguish whether the period-doubling bifurcation is sub- or supercritical. To clarify this matter for the left stability boundary of SyC, in Fig. 5(B) we use multiple continuations and simulations of the model. We found that in a small vicinity on the left of the critical boundary the bifurcation diagram is rather complex. In fact, some part of this boundary corresponds to subcritical and some part corresponds to supercritical period-doubling bifurcation. In the case of supercritical bifurcation, on crossing the boundary, the stable synchrony limit cycle becomes unstable and a stable double-period cycle appears. This cycle is stable in a very small vicinity of the period-doubling boundary and becomes unstable via pitchfork bifurcation. We do not report complex bifurcations in this small vicinity on the left of the boundary and indicate that this boundary relates to the subcritical period-doubling bifurcation. Thus, if we do not consider a small region near this boundary, then the only stable attractor is the SwC. A similar remark is valid for the upper part (from the LPD point) of the left critical boundary in Fig. 5(A).

### Study of the Initiation Space

**Fig. 8:**
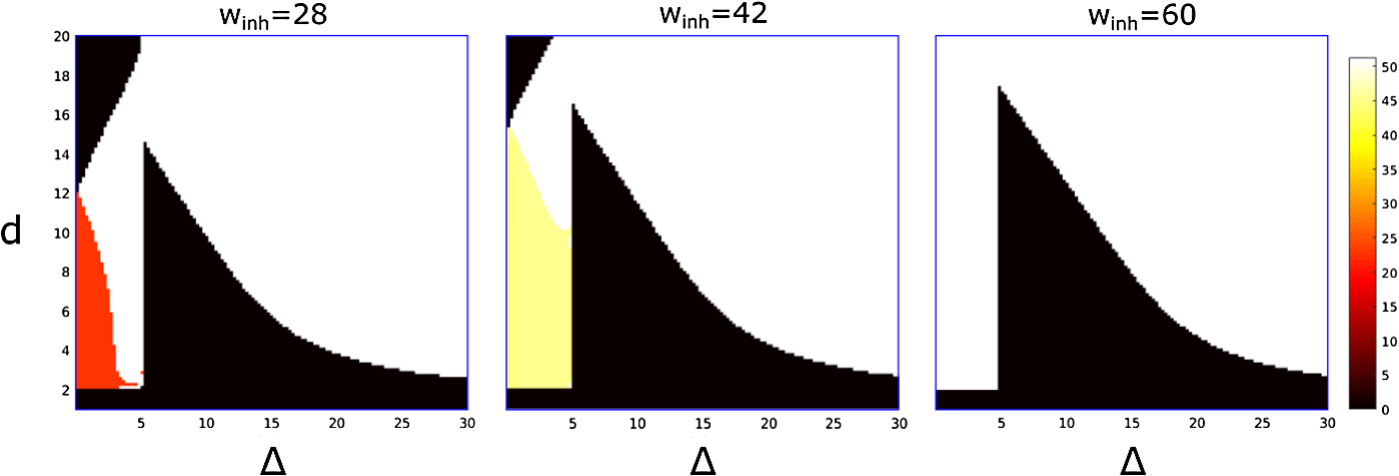
Stable attractors of the reduced model at varying initiation parameters ($\Delta,d$) with fixed $w_{\mathrm{ampa}} =10$ nS and $w_{\mathrm{ndma}} =10$ nS. We show three different values of $w_{\mathrm{inh}} =28,\ 42 $ and 60 nS (title of each subplot). These values correspond to all the possible combinations of stable attractors of the system shown in Fig. 6. Each coloured region identifies the initialisation parameters ($\Delta,d$) that converge to a stable limit cycle, or convergence to the resting state (black regions, fixed point). In the case of convergence to a limit cycle, the colour represents the period of the attractor. The orange region in the case $w_{\mathrm{inh}} =28$ nS identifies the initial conditions where the system converges to stable synchrony, the yellow region in the case $w_{\mathrm{inh}} =42$ nS corresponds to convergence to the stable 2-synchrony, while the white regions correspond to convergence to stable swimming

In this section we study how the dynamical mode depends on initiation parameters. We consider a grid of two parameter pairs: initiation time difference Δ and duration *d*. The amplitude initiation parameter is a fixed value $A=0.01$. The rectangular area of the initiation space ($0\leq\Delta\leq30$ and $0\leq d \leq20$) is covered by a grid of *n* by *n* nodes uniformly spaced ($n=128$). For each node in the grid, we initiate the system dynamics. We run the simulations for a long time (3 simulated seconds) so that the trajectory approaches an attractor. This attractor can be either a limit cycle or a fixed point (resting state). In the case of a limit cycle, we calculate the period of oscillations. Figure 8 shows the result of simulations with fixed parameters $w_{\mathrm{nmda}} = w_{\mathrm{ampa}} =10$ nS for different values of $w_{\mathrm{inh}}$ (28,42,60 nS). A black pixel at position ($\Delta,\ d$) means that initiation with these parameters results in a fixed point (period 0). If the initiated trajectory tends to a limit cycle, then we discriminate the limit cycle by computing its period. Parameter value $w_{\mathrm{inh}} =28$ nS corresponds to regions of coexistence of stable SwC and stable SyC. Parameter value $w_{\mathrm{inh}} =42$ nS corresponds to regions of coexistence of stable SwC and the stable 2-SyC (type (2) cycle). For $w_{\mathrm{inh}} =60$ nS, only SwC is stable. These particular values for $w_{\mathrm{inh}}$ have been selected using the bifurcation diagram that was described in Sect. 3.4. This diagram allows us to explore the initiation space for all the stable attractors of the system. In all cases the largest region of initiation space corresponds to stable swimming (period of ∼50 ms), but for some parameter values there is also a relatively small region where simulations converge to either synchrony (period of ∼20 ms) or the double-period synchrony cycle (period of ∼45 ms).

In Fig. 8 all three panels include a vertical boundary near $\Delta =5$ ms. This boundary separates the white swimming region (or double-synchrony yellow region in the middle panel) from the black rest state region. In fact, the position of this boundary is determined by the time difference between first spikes of the left-dIN and left-cIN which we denote by *μ* ($\mu\ \approx 5$ ms).

Indeed, if the value of parameter *d* is limited and the time interval $\Delta>\mu$, then stimulation of the right dIN will not generate a spike because at the time of stimulation the right dIN will be under strong inhibition. Therefore, the system will move to the rest state. To explain the right boundary of the black rest state region, we note that after some time the inhibition of the right dIN becomes weaker. Therefore, for some appropriate values of parameter Δ (for a fixed moderate value of parameter *d*), stimulation of the right dIN will overcome the inhibition, the right-dIN will spike and the system will converge to swimming.

In case of a short delay $\Delta<\mu$, the right-dIN will spike because the stimulation of this dIN precedes the inhibition from the left-cIN. This dIN spike will trigger a spike in the right-cIN and it will lead to rhythmic activity. This rhythmic spiking can be either double-synchrony (yellow colour region in the middle panel) or swimming (white colour area).

If $d<2$ ms the injected currents of the initiation procedure are too short to activate either of the two dINs, and the system converges to the rest state (small black rectangular region in all three panels).

### Interpretation of Bifurcation Diagrams in Terms of Experimental Recordings

In this section, we speculate on how our study of the reduced model can explain the long-lasting synchronous activity seen in some biological experiments. First, we find that patterns of spiking activity recorded in experiments following skin stimulation are very similar to spiking patterns and voltage traces generated by the reduced model. Second, our study of bifurcation enables us to formulate hypotheses on the existence of the synchrony mode and bi-stability regime where both swimming and synchrony modes co-exist for the same parameter values.

We show that the system’s bifurcations and the particular initiation procedure used play important roles in explaining long-lasting synchronous activity and a subsequent transition to swimming. To explain this, we consider model parameters near the bifurcation points shown in Fig. 6.

**Fig. 9:**
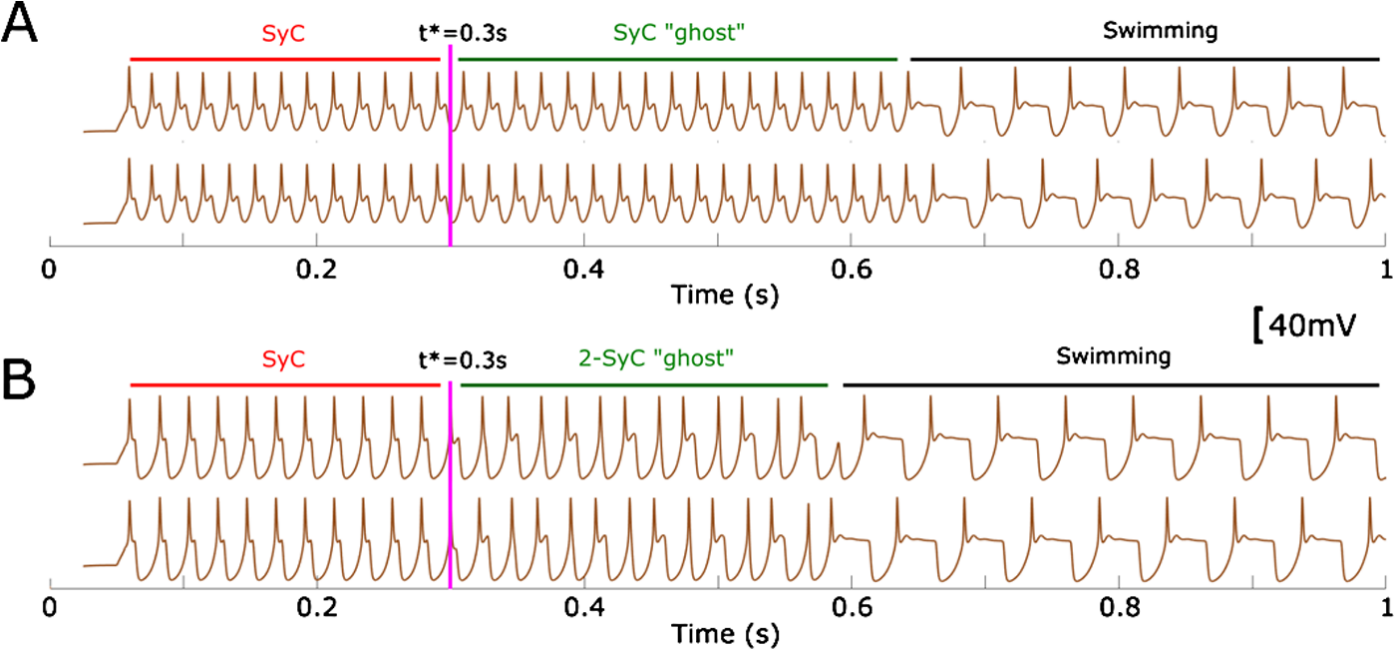
Transition from synchrony to swimming. Plot of dINs’ voltage recordings at varying time shows synchronous activity before the dynamics are locked into synchronous (**A**) and double-synchronous regimes (**B**) before then converging to the swimming mode. In both (**A**) and (**B**) initiation parameters are set to $\Delta=0$, $d=6$ and $A=0.04$. At time $t^{*} =0.3$ s the system is integrated starting from a perturbed initial point. This point is obtained by adding a normally distributed vector of numbers with equal variance $\sigma=10^{-3}$ to each variable at time $t^{*}$. Values of synaptic strengths are $w_{\mathrm{ampa}} =10$, $w_{\mathrm{nmda}} =10$, $w_{\mathrm{inh}} =22.2$ in case (**A**) and $w_{\mathrm{inh}} =60$ in case (**B**)

*Synchrony (double-synchrony) to swimming transitions.* In Fig. 9(A), the selected parameter values correspond to the orange region of the bifurcation diagram in Fig. 6(B) (ZOOM 1). For any parameter value inside this region, the SyC is globally unstable, but it is stable inside the symmetry manifold $Y_{k}^{+}$. The initiation parameter value $\Delta=0$ means that the orbit starts and remains on the invariant symmetry manifold $Y_{k}^{+}$. Although SyC is unstable, the trajectory converges to this limit cycle. At time $t^{*} =0.3$ s we slightly perturb the last point of the trajectory by adding a normally distributed vector with zero mean and variance $\sigma= 10^{-3}$. We then restart the system integration from the perturbed point. The perturbed point does not belong to the invariant symmetry manifold; therefore, trajectory diverges from the manifold and tends to SwC. The transitional period from the vicinity of the manifold to SwC is long because the value of $w_{\mathrm{inh}}$ is close to the subcritical pitchfork bifurcation (critical parameter value is $u_{4}$ in Fig. 6(B), ZOOM 1).

The transition time spent near the “ghost” of the stable synchrony cycle tends to infinity as $w_{\mathrm{inh}}$ tends to the critical value of pitchfork bifurcation. This effect is valid for any parameter in the orange region $w_{\mathrm{inh}} \in( u_{5}, u_{4} )$ in Fig. 6(B), ZOOM 1. Although both swimming and resting states are stable, for $u_{1} \leq w_{\mathrm{inh}} \leq u_{5}$ the system converges to the resting state under the initiation procedure with parameter values used in Fig. 9 ($\Delta=0$, $d=6$ and $A=0.04$). These parameters correspond to the orbit initiation inside the symmetry manifold $Y_{k}^{+}$. For parameter values $u_{1} \leq w_{\mathrm{inh}} \leq u_{5}$, the SyC is repulsive inside $Y_{k}^{+}$ (in Fig. 9(B), ZOOM 1 both unstable cycles shown by blue and red dotted lines belong to the symmetry manifold); therefore, the orbit stays inside the symmetry manifold and converges to the resting state. By multiple simulations we confirmed that the basin of attraction for the resting state is large, therefore, small perturbations ($\sigma<0.1$) cannot move the system to another attractor.

In Fig. 9(B), the selected parameter values are inside the light blue region in Fig. 6(A) and Fig. 6(B) (ZOOM 3) corresponding to $w_{\mathrm{inh}} \geq u_{8}$. The critical parameter value $w_{\mathrm{inh}} = u_{8}$ corresponds to a fold bifurcation, and the stable 2-SyC disappears. Near this bifurcation on the right side ($w_{\mathrm{inh}} = u_{8}$) a ghost of this limit cycle exists. We start the dynamics with initiation parameter $\Delta=0$, and the trajectory converges to unstable SyC. At time $t^{*} =0.3$ s we perturb the last point of the trajectory by adding a normally distributed random number to all system variables (the mean is zero and the variance $\sigma=10^{-3}$). Integration from the perturbed point results is a long transitional period near the ghost of 2-SyC cycle and convergence to the SwC. This long transition can be reproduced for all parameter values $w_{\mathrm{inh}} \in( u_{8},70)$ in the light blue region of Fig. 6(A). Remarkably, $w_{\mathrm{inh}}$ does not need to be too close to the bifurcation point to obtain long-lasting transitions, provided values of the perturbation parameter *σ* are small. For example, with $w_{\mathrm{inh}} =70$ nS and $\sigma=0.01$, we can still obtain a ∼1 s transition time.

In addition, this study of bifurcation provides insights into explanation of some recordings from CPG neurons. Figure 6(C) in [12] shows that under depolarizing current injection, dINs can fire an additional spike at approximately half the swimming period and initiate synchrony. The voltage recordings of these neurons look very similar to the 2-SyC “ghost” part of trajectory in Fig. 9(B). It is not clear from the experiment why “mid-cycle spikes” appear in the recordings. Our study provides an explanation of this experimental observation.

*Distributions of the duration of the synchrony (double-synchrony) bouts*. Experimental findings show that the time of transition from synchrony (double-synchrony) to swimming can be distributed in a wide range from 100 to 1000 ms [12]. To study how this time of transition depends on the system perturbation, we add white noise to the deterministic model (11). The following continuous stochastic process describes the model with noise:

12 $$ du=f ( u ) \boldsymbol{\cdot}\,dt+\phi\boldsymbol{\cdot}\,d W_{t},\quad u ( t ),f ( u ), W_{t} \in \mathbb{R}^{2k}, $$

where $u ( t )$ is the solution (12), $f ( u )$ is the vector of right-hand side, $W_{t}$ represents a standard vector of independent Weiner processes and *ϕ* is a small parameter ($\phi=0.01$). We use Eurler–Maruyama integration to compute the numerical solution of (12), and we find that in the large majority of random simulations this solution shows transitions from synchrony (double-synchrony) like that in Figs. 9(A), (B) (with the same parameter values as Fig. 9).

**Fig. 10:**
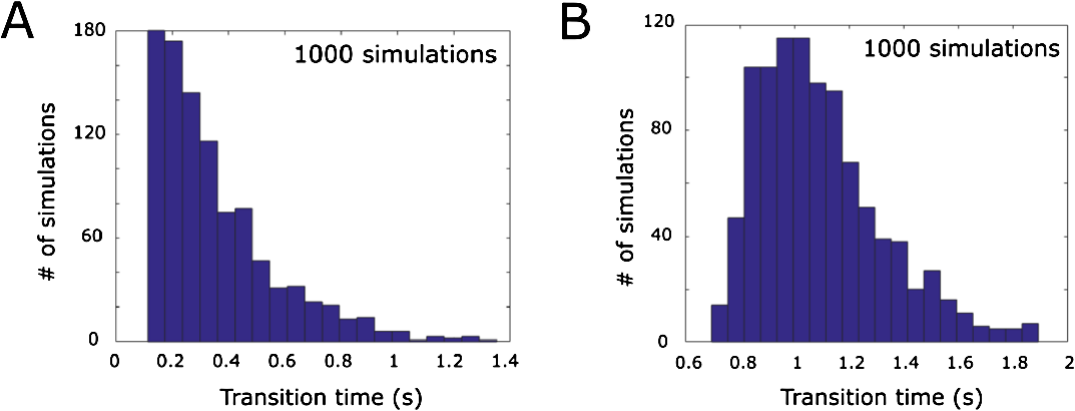
Distribution of times spent in the synchrony (double-synchrony) transition in the system with noise. (**A**) and (**B**) show the histogram of times spent in synchronous state before switching to swimming. The selected parameter values of (**A**) and (**B**) are the same as the ones used in Fig. 7(A) and (B), respectively

We run 1000 simulations with independent random seeds. For each simulation we integrate the system for 2 s and detect the time of switching from synchrony (double-synchrony) to swimming. In 97% of cases the system demonstrates a transition from synchrony to swimming and in 3% the system switches to the resting state. Figure 10(A) shows the histogram of switching time from synchrony to swimming. It is clear from this figure that the time of transition from synchrony to swimming is variable and the range of transition times is compatible with those observed in experiments (see Fig. 2 in [12]). Considering the transition from double-synchrony to swimming, we find that for any random seed the system demonstrates transitions from double-synchrony to swimming. Figure 10(B) shows the histogram of switching time from double-synchrony to swimming.

**Fig. 11:**
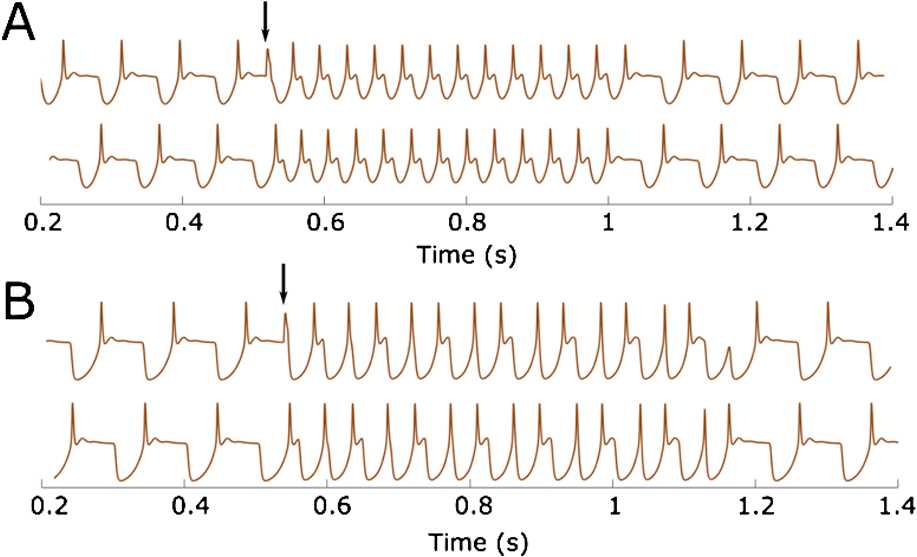
Plot of dIN voltage dynamics showing transitions from swimming to synchrony (**A**) or double-synchrony (**B**), and back to swimming. In both (**A**) and (**B**) a brief step current (0.45 nA, 5 ms) is manually injected to the left dIN at the time of right dIN firing (black arrows). Parameter values used to obtain (**A**) and (**B**) are the same as the ones used in Fig. 9(A) and (B), respectively, except that $\Delta=50$

*From swimming to synchrony (double-synchrony) and back*. We show that the reduced model can reproduce transitions from swimming to synchrony and switch back to swimming similarly to what is observed in experimental recordings [12]. To initiate synchrony from swimming in physiological experiments, one side is stimulated at the middle of the swimming period. We mimic these experiments to initiate synchrony keeping parameter values as in Fig. 9(A). Figure 11(A) shows injection of a brief positive step current to the left dIN in the middle of the swimming cycle (shown by an arrow). This injection evokes an additional spike which is nearly synchronous with the firing of the right dIN. This additional spike starts a long-lasting synchrony bout before switching back to swimming, like in experimental recording [12]. Similarly, Fig. 11(B) shows that mid-cycle stimulation (shown by an arrow) of the left dIN during the swimming mode can evoke a long-lasting bout of double-synchrony oscillations.

### Breaking Symmetry Does Not Change the Stability of Swimming and Synchrony

In this section we analyse the effect of symmetry-breaking in the reduced model. To break the $\mathbb{Z}_{2}$-symmetry of the system, we slightly perturb the maximal conductance of all ion channels by adding normally distributed random variable with mean equal to zero and standard deviation 0.1. This perturbation is applied to all neurons using a different random seed for each perturbed parameter. All other parameters of neuronal activity and synaptic transmission are identical. As a result of this perturbation, we break the symmetry of the reduced model and consider a non-symmetrical system (NSS).

**Fig. 12:**
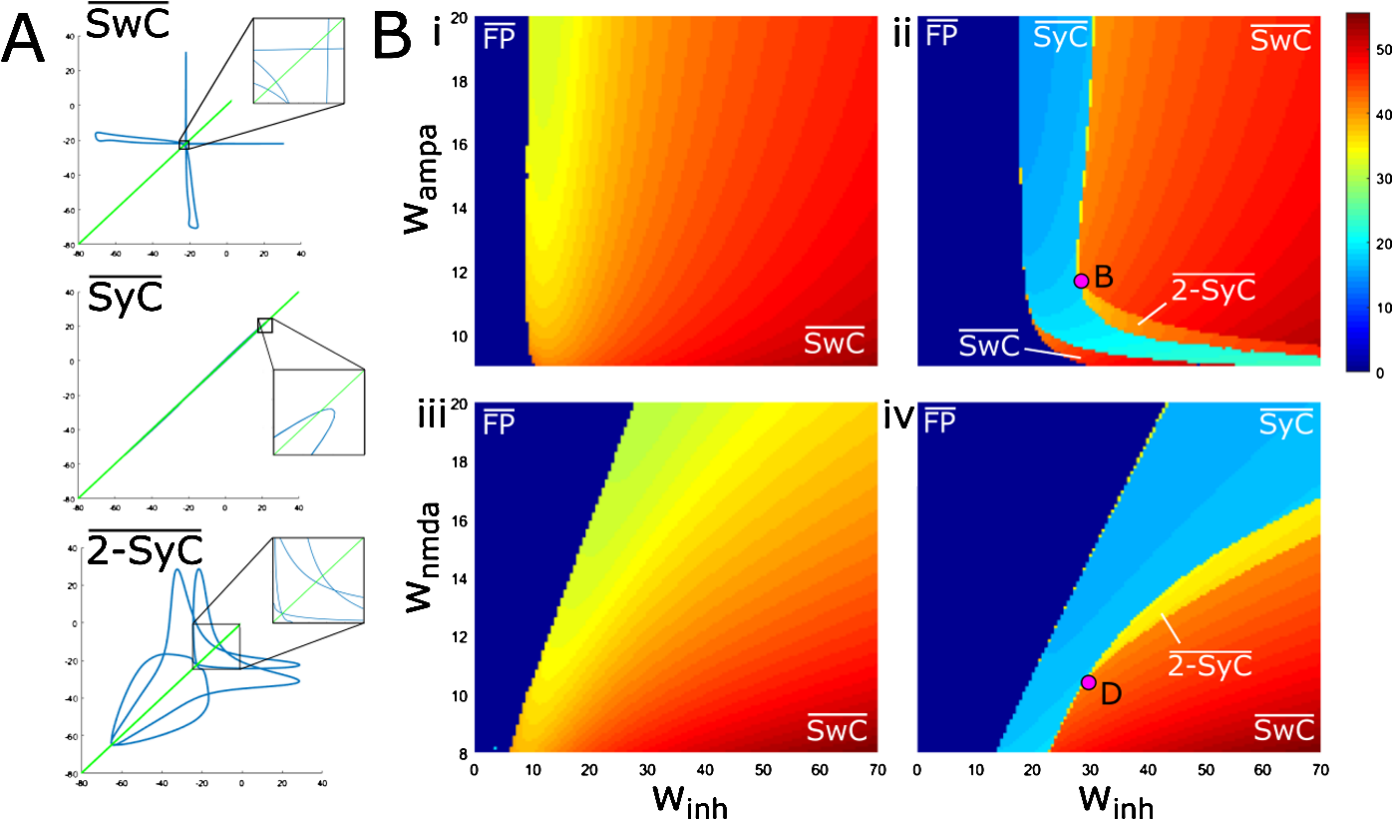
Stability of the attractors of after symmetry breaking (**A**) Projection of the three stable limit cycles (SwC̅, SyC̅ and $\overline{2-\mathrm{SyC}}$) to the phase plane of dINs voltages and zoom of selected regions (black boxes). The green diagonal line shows the loss of mid-line symmetry of the stable limit cycles. The vector of parameters ($w_{\mathrm{inh}}, w_{\mathrm{ampa}}, w_{\mathrm{nmda}},\Delta,d$) used for the SwC̅ case are ($60,10,10,100,6$), for the SyC̅ case are ($25,12,10,10^{-4},6$), and for the $\overline{2-\mathrm{SyC}}$ case are ($40,10,10,10^{-4},6$) (**B**) Period of the attracting limit cycle found by numerical simulation at varying ($w_{\mathrm{inh}}, w_{\mathrm{ampa}}$) and fixed $w_{\mathrm{nmda}} =10$ nS in cases (i)–(ii) and at varying ($w_{\mathrm{inh}}, w_{\mathrm{nmda}} $) and fixed $w_{\mathrm{ampa}} =12$ nS in cases (iii), (iv). Initiation parameters for cases (i)–(iii) are $\Delta=50 $ and $d=6$, while for cases (ii)–(iv) are $\Delta= 10^{-4}$ and $d=6$

Studying the bifurcations of the symmetrical system (SS) under variation of two parameters, we find that there are three stable limit cycles: SyC, SwC and 2-SyC (Fig. 7). Simulations of the NSS show that the three stable limit cycles (SyC̅, SwC̅, $\overline{2-\mathrm{SyC}}$) exist and have a shape and pattern of firing very similar to the corresponding cycles for the SS. Figure 12(A) shows projections of stable limit cycles of NSS to the plane of left-right dIN voltages for three stable limit cycles. For each projection, zooming into part of the phase portrait helps to visualise a small “imperfection” of the limit cycle and deviation from the diagonal. This figure clearly demonstrates that three stable cycles are not symmetrical.

To find the stability regions for the stable limit cycles SyC̅, SwC̅, $\overline{2-\mathrm{SyC}}$ of NSS under variation of two parameters ($w_{\mathrm{inh}}, w_{\mathrm{ampa}} $) and $( w_{\mathrm{inh}}, w_{\mathrm{nmda}} )$, we use massive simulations of the perturbed reduced model. We consider the same region of parameters as in Fig. 5 and with the uniform $n\times n$ grid ($n=128$). For each node of the grid, we simulate the same NSS using the same seed for the random number generator and simulate trajectory for long times (30 sec) enabling convergence to the limit cycle attractor. Similar to the SS case, we find that a trajectory approaches either a stable limit cycle or a fixed point. In the case of a limit cycle, we compute the period of oscillation. Figures 12(B)(i), (ii) and Figs. 12(B)(iii), (iv) show the results of these computations under variation of ($w_{\mathrm{inh}}, w_{\mathrm{ampa}} $) and ($w_{\mathrm{inh}}, w_{\mathrm{nmda}} $), respectively. All simulation parameters used to simulate trajectories and compute each period are reported in the caption of Fig. 12.

In Fig. 12(B) we use colour coding to show the period of each stable attractor for the pair of parameters ($w_{\mathrm{inh}}, w_{\mathrm{ampa}} $) and ($w_{\mathrm{inh}}, w_{\mathrm{nmda}} $). Figure 12(B)(i) shows the stability regions for two attractors: a fixed-point attractor (dark blue) and the SwC̅ attractor (yellow-red colours indicating periods in the range 35–50 ms). It is clear from the figure that the period of swimming increases with increasing $w_{\mathrm{inh}}$ for any fixed value of $w_{\mathrm{ampa}}$. It is interesting to note that the separation line between these two regions matches the black line (TR^−^) in Fig. 5(A) corresponding to the subcritical torus bifurcation of the symmetrical system.

Similarly, in Fig. 12(B)(iii) there are also two different regions (colour coded as in part A). In this case, the period of swimming increases with increase of $w_{\mathrm{inh}}$ for any fixed value of $w_{\mathrm{nmda}}$. The separation line between these two regions matches the black line (TR^−^) in Fig. 5(B) again corresponding to the subcritical torus bifurcation of the symmetrical system.

Figures 12(B)(ii) and (iv) show the results of simulations with initiation parameters corresponding to the synchrony mode (SyC̅ and $\overline{2-\mathrm{SyC}}$). Dark blue again means a trajectory that converges to the fixed-point attractor. The light blue area shows the stability region of SyC̅. This region and its boundaries match the region and boundaries of the stable synchrony region in the case of SS (Fig. 5(B)).

In Fig. 12(B)(ii), the left boundary of the SyC̅ stability region relates to two transitions from the synchrony mode: (1) transition to the fixed point and (2) transition to the swimming mode (dark red area). Both transitions match the bifurcation lines in Fig. 5(A). The right boundary of the SyC̅ stability region also relates to two transitions: (1) The first is the transition to the swimming mode (red area). The boundary of this transition, up from point *B* in Fig. 12(B)(ii), matches the subcritical period-doubling bifurcation line in Fig. 5(A). (2) The second is the transition to the double-period synchrony mode $\overline{2-\mathrm{SyC}}$ (yellow-brown area). The boundary of this transition, down-right from point *B* in Fig. 12(B)(ii), fits well to the supercritical period-doubling bifurcation line in Fig. 5(A). This region of stability of the doubled-period $\overline{2-\mathrm{SyC}}$ cycle is narrow with transitions to the swimming mode. Remarkably, our simulations show a stability region of $\overline{2-\mathrm{SyC}}$ which was not found by study of bifurcations.

In Fig. 12(B)(iv) the left boundary of the SyC̅ stability region (light blue area) relates to transitions from the “synchrony” mode to the fixed-point attractor (dark blue area). The right boundary of the SyC̅ stability region relates to two transitions: (1) Transition to double-period synchrony mode $\overline{2-\mathrm{SyC}}$ (yellow area). The boundary of this transition, up from point *D* in Fig. 12(B)(iv), fits well to the supercritical period-doubling bifurcation line in Fig. 5(B). (2) Transition to the swimming mode. The boundary of this transition, down-left from point *D* in Fig. 12(B)(iv), fits well to the subcritical period-doubling bifurcation line in Fig. 5(B). It is interesting to note that again the simulations show the region of stability of the double-period cycle $\overline{2-\mathrm{SyC}}$ (the narrow yellow strip with transitions to the swimming mode) which was not found by study of bifurcations.

Thus, we conclude that symmetry-breaking by a small perturbation of maximum conductance parameters leads to a minor change of limit cycle stability boundaries. Stability boundaries of NSS fit well to bifurcation lines of the symmetrical system. In addition, simulation results help to clarify the stability of dynamical regimes in the vicinity of codimension-two bifurcation within the complex structure of the bifurcation diagram.

## Discussion

### Summary of Main Findings

In this study we have developed a reduced model of the core neuronal elements of the circuit that drives swimming the hatchling *Xenopus* tadpoles. We have used bifurcation theory to provide a mathematical description of two main oscillatory modes under variation of key parameters of this model. These modes of anti-phase and in-phase oscillations correspond to swimming and synchrony patterns of spiking activity, respectively. Both of these spiking patterns can be observed in physiological experiments where neurons typically fire in alternation (in swimming mode), but can also (occasionally) fire synchronously at half the swimming cycle period (synchrony mode). Bifurcation analysis has shown the boundaries of the region in a space of two parameters where the stable synchrony regime exists. This synchrony stability region lies within a much larger region corresponding to stable swimming. Therefore, the intersection of these two regions is a region of bi-stability. We conclude that the same pattern generator circuit can support both swimming and synchrony. A crucial factor in determining which pattern is expressed is the way in which the oscillation is initiated. In addition to swimming and synchrony, we have also described a further stable spiking pattern which we term double-period synchrony.

### Significance of Using the Reduced Model

We study a reduced model, which can be considered the result of “averaging” of the biologically realistic functional model of the tadpole spinal cord [34]. Specifically, we ignore parts of the functional model corresponding to sensory pathways and consider only the key part of the tadpole’s CPG circuit, as derived from biological measurements and designed to capture the important details. The two neuron types included (dIN and cIN) are the core of the CPG and the model specification for each is based on available knowledge of their real biological characteristics, including ionic channel currents [28]. The reduction is achieved by minimising the number of neurons considered, leaving just two neurons in each half-centre (each side of the body): one excitatory (dIN) and one inhibitory commissural (cIN). Of course, an even smaller circuit constituted by two mutually inhibitory neurons with PIR can generate an anti-phase swimming [47]. However, the mechanism of tadpole CPG functioning is different. In [5] there is a comparison between the tadpole and clione CPG circuits (the clione CPG is believe to work as a chain of two mutually inhibitory neurons with PIR).

The essential connectivity between these neurons is maintained. Models of synaptic connections are also biologically realistic; for example, the glutamatergic transmission from dINs acts at separate NMDA and AMPA type receptors with different properties [48]. One addition made to the model is feedback self-excitation of each dIN to compensate for the mutual excitation between separate dINs in the same half-centre that is lost when reducing the model to a single neuron per type. As a result, the voltage dynamics of the model shows patterns of neuron activity that are very like those seen in real recordings and previous CPG modelling [12, 27], and show characteristic features of spike dynamics, such as post-inhibitory rebound [47]. The reduced model therefore encapsulates the core features of the full circuit.

Model reduction is essential for allowing a detailed bifurcation analysis of the system. Different approaches for reducing highly complex neuronal systems have been proposed and have been applied to the study of bifurcations in CPG networks [49–52]. These approaches tend to reduce the number of differential equations describing neuronal properties by considering simplified neuron models, non-spiking neuron models or phase/amplitude reductions [23, 53–57]. A further simplification made in CPG circuits is the reduction of the number of synaptic interactions by considering the minimal number of connections describing the circuit “building blocks” [7, 58]. Our approach is different: we do not minimise the number of equations describing the dynamics of single neurons, but we reduce the number of neurons and connections, keeping the important biological properties of spike generation and synaptic interactions. Even with the significant reduction in scale relative to the whole swimming circuit, the dynamical system describing the neuronal activity was still relatively large and included 34 variables. It is a challenging problem to study bifurcations of limit cycles in a dynamical system of such high dimension. For instance, it is known that the numerical algorithms for continuation in the case of high dimensional systems are not reliable near the critical parameter value of period-doubling bifurcation. However, using AUTO, and after adjustment of multiple numerical parameters, it was possible to continue the limit cycles and detect bifurcations up to codimension two. Our studies have been restricted to continuation of limit cycles corresponding to swimming and synchrony. The swimming (synchrony) limit cycle is characterised by anti-phase (in-phase) oscillations of equivalent neurons on opposite body sides.

### Simplified Initiation and the Significance of the Pattern of Initiation

One feature known to be over-simplified in the most recent model of the full swimming circuit [34] is the mechanism for initiating rhythm following a brief stimulus. Fundamentally, the requirement is simply that oscillations on both sides (in each half-centre) need to be initiated and coordinated. In the reduced model, the process is also much more simplified: the triggering stimulus to each side is sufficient to initiate oscillation and this allows us to focus attention on the effect of timing differences between stimuli to the two sides. We have illustrated effects of changing the stimulus duration, but we do not consider these further here. Running multiple simulations showed that stimuli are much more likely to initiate swimming than synchrony. To produce synchrony, timing differences between stimuli to the two sides must be very small. This would suggest that, in biological terms, an initiation mechanism that avoids such near-simultaneous activation of the two sides (see below) is required.

### Stable States and Symmetry

Our study of bifurcations provides new insights into the mechanisms of CPG spike production. This study reveals three spiking patterns of neuronal activity corresponding to swimming, synchrony and double-period synchrony, each of which is stable in some area of the parameter space. The largest area of stability corresponds to the swimming pattern. In swimming, there is typical slow voltage decay after each dIN spike followed by a deep inhibition which leads to a subsequent spike by post inhibitory rebound. Spiking in the equivalent dIN neuron in the opposite half-centre is exactly in anti-phase. The stable synchrony pattern is characterised by simultaneous spiking of equivalent neurons on the two sides and with a period of half that seen in swimming. The third stable spiking pattern revealed in our analysis is what we have termed double-period synchrony. The period of this mode is close to the swimming period and the spiking pattern also resembles swimming, but with an additional spike with slightly different shape at mid-cycle, giving an appearance superficially like that of synchrony. However, in double-period synchrony, spiking of equivalent neurons on the two sides is near-synchronous rather than synchronous. Alternate spikes in the dIN in each half-centre occur just ahead of and then just behind the dIN spike in the opposite half-centre. Like swimming and synchrony, a pattern resembling double-period synchrony has also been described experimentally (see below).

The analysis of bifurcations in the reduced model takes into account the left-right half-centre symmetry. Because of this symmetry, we detect some properties that are exclusive of symmetric dynamical systems [3, 46]. For example, there are two types of cycles originating from the period-doubling bifurcation of the symmetry cycle. Of the two types of double-period synchrony cycles, one is unstable and left-right symmetrical, while another is stable and its right-half variables are symmetrical to the left-half variables shifted by half-period. Our results on bifurcations are not limited to the symmetric system, but extend to systems where the symmetry is broken by including a small perturbation to some equation parameters. Trivially, all the bifurcations change to non-symmetrical ones (for example, pitchfork becomes fold).

### Biological Links and Significance

As outlined above, our reduced model displays three stable spiking patterns. Remarkably, these three characteristic patterns correspond well to experimental recordings of spiking activity from spinal cord neurones.

Of these, swimming is the most biologically relevant: it is the pattern of activity shown by the CPG neurons that drive muscles to provide the main behavioural response in tadpoles. In experiments, long-lasting swimming is initiated by a brief sensory stimulus (touch) to the head or trunk skin [34, 41]. The spiking patterns of dIN and cIN neurones in the swimming mode represent the typical activity of CPG neurons. Remarkably, our analysis revealed that the largest area of parameter space is occupied by stable swimming.

Synchrony is seen in occasional experimental recordings, where it can last for several hundred milliseconds (perhaps 10–15 cycles) before returning to swimming [12]. The synchrony pattern occupies a substantial area of parameter space; however, it lies within the area for swimming, hence it is an area of bi-stability. Bifurcation analysis shows that both types of stability boundary of the synchrony cycle correspond to subcritical bifurcations (pitchfork and period-doubling bifurcation lines). Therefore, the loss of stability by the synchrony cycle will result in a change of dynamical mode, particularly from synchrony to swimming, just as observed experimentally. Like experimentally recorded neurons, this modality change can take several seconds if model parameters are near the bifurcation points which determine the loss of stability for the synchrony cycle.

Although synchronous activity in the limbs will become a characteristic of the tadpole as it nears metamorphosis to the adult [11], there is no evidence that the synchrony pattern modelled here has any function in young tadpoles. It is more likely, therefore, that the priority is to avoid the expression of this pattern. Analysis of the initiation parameters in the reduced model suggests that the important factor here is to minimise the likelihood of oscillations on the two sides being initiated within a very short time delay, since such short delays make synchrony more likely. We should predict that the initiation circuitry in the tadpole will be constructed so as to introduce delays that ensure activation on the two sides while avoiding co-activation.

We have concentrated on analysing the stability of limit cycles corresponding to swimming and synchrony. However, we find that there are several unstable limit cycles, which should be also included into consideration for clarity of the multiple interlinked bifurcations. Some of these unstable cycles are shown on our bifurcation diagrams for completeness of the analysis. Moreover, we found one more stable mode—double-period synchrony. As with synchrony, there is no evidence for a biological role for this regime. Double synchrony activity can be observed experimentally, for example by injecting depolarising current into a dIN, or this regime can spontaneously occur during swimming. From the biology point of view, the regime corresponding to the double-synchrony in the model appears if the spiking of two dINs on the opposite body sides is not perfectly synchronised, and the jittered cIN inhibition does not suppress dIN spiking on either side [12]. Remarkably, the spiking pattern named double-synchrony in the reduced model perfectly reproduces this experimental finding and the shape of dIN-cIN voltages is very similar to experimental recordings (Fig. 7(A)).

## Electronic Supplementary Material

Below is the link to the electronic supplementary material.

Supplementary material (DOCX 89 kB)

## Abbreviations

CPG: central pattern generator
dIN: excitatory neuron
cIN: inhibitory neuron
PIR: post inhibitory rebound
SyC: synchrony limit cycle
SwC: swimming limit cycle
2-SyC: double-period synchrony limit cycle
LP: fold of limit cycles
PD: period-doubling bifurcation
PFK: pitchfork bifurcation
TR: Neimark–Sacker bifurcation
LPD: Fold-flip bifurcation

## References

[1] Roberts A, Soffe SR, Wolf ES, Yoshida M, Zhao FY (1998). Central circuits controlling locomotion in young frog tadpoles. Ann NY Acad Sci.

[2] Grillner S, Wallén P, Saitoh K, Kozlov A, Robertson B (2008). Neural bases of goal-directed locomotion in vertebrates—an overview. Brains Res Rev.

[3] Golubitsky M, Stewart I, Buono PL, Collins JJ (1999). Symmetry in locomotor central pattern generators and animal gaits. Nature.

[4] Marder E, Bucher D (2001). Central pattern generators and the control of rhythmic movements. Curr Biol.

[5] Arshavsky YI, Orlovsky GN, Panchin YV, Roberts A, Soffe SR (1993). Neuronal control of swimming locomotion: analysis of the pteropod mollusc Clione and embryos of the amphibian Xenopus. Trends Neurosci.

[6] Dimitrijevic MR, Gerasimenko Y, Pinter MM (1998). Evidence for a spinal central pattern generator in humans. Ann NY Acad Sci.

[7] Marder E, Calabrese RL (1996). Principles of rhythmic motor pattern generation. Physiol Rev.

[8] Ijspeert AJ (2008). Central pattern generators for locomotion control in animals and robots. Neural Netw.

[9] Grillner S (2006). Biological pattern generation: the cellular and computational logic of networks in motion. Neuron.

[10] Eisenhart FJ, Cacciatore TW, Kristan WB (2000). A central pattern generator underlies crawling in the medicinal leech. J Comp Physiol A.

[11] Combes D, Merrywest SD, Simmers J, Sillar KT (2004). Developmental segregation of spinal networks driving axial-and hindlimb-based locomotion in metamorphosing Xenopus laevis. J Physiol.

[12] Li WC, Merrison-Hort R, Zhang HY, Borisyuk R (2014). The generation of antiphase oscillations and synchrony by a rebound-based vertebrate central pattern generator. J Neurosci.

[13] Dickinson PS, Mecsas C, Marder E (1990). Neuropeptide fusion of two motor-pattern generator circuits. Nature.

[14] Briggman KL, Kristan WB (2006). Imaging dedicated and multifunctional neural circuits generating distinct behaviors. J Neurosci.

[15] Briggman KL, Kristan WB (2008). Multifunctional pattern-generating circuits. Annu Rev Neurosci.

[16] Roberts A, Li WC, Soffe SR, Wolf E (2008). Origin of excitatory drive to a spinal locomotor network. Brains Res Rev.

[17] Roberts A, Li WC, Soffe SR (2010). How neurons generate behaviour in a hatchling amphibian tadpole: an outline. Front Behav Neurosci.

[18] Kahn JA, Roberts A (1982). The central nervous origin of the swimming motor pattern in embryos of Xenopus laevis. J Exp Biol.

[19] Kahn JA, Roberts A (1982). Experiments on the central pattern generator for swimming in amphibian embryos. Philos Trans R Soc Lond B, Biol Sci.

[20] Soffe SR, Clarke JD, Roberts A (1984). Activity of commissural interneurons in spinal cord of Xenopus embryos. J Neurophysiol.

[21] Roberts A, Dale N, Soffe SR (1984). Sustained responses to brief stimuli: swimming in Xenopus embryos. J Exp Biol.

[22] Roberts A, Tunstall MJ (1990). Mutual re-excitation with post-inhibitory rebound: a simulation study on the mechanisms for locomotor rhythm generation in the spinal cord of Xenopus embryos. Eur J Neurosci.

[23] Molkov YI, Bacak BJ, Talpalar AE, Rybak IA (2015). Mechanisms of left-right coordination in Mammalian locomotor pattern generation circuits: a mathematical modeling view. PLoS Comput Biol.

[24] Wolf E, Soffe SR, Roberts A (2009). Longitudinal neuronal organization and coordination in a simple vertebrate: a continuous, semi-quantitative computer model of the central pattern generator for swimming in young frog tadpoles. J Comput Neurosci.

[25] Laing AR, Carson CC (2002). A spiking neuron model for binocular rivalry. J Comput Neurosci.

[26] Li WC, Soffe SR, Wolf E, Roberts A (2006). Persistent responses to brief stimuli: feedback excitation among brainstem neurons. J Neurosci.

[27] Soffe SR, Roberts A, Li WC (2009). Defining the excitatory neurons that drive the locomotor rhythm in a simple vertebrate: insights into the origin of reticulospinal control. J Physiol.

[28] Dale N (1995). Experimentally derived model for the locomotor pattern generator in the Xenopus embryo. J Physiol.

[29] Winlove AI, Roberts A (2012). The firing patterns of spinal neurons: in situ patch-clamp recordings reveal a key role for potassium currents. Eur J Neurosci.

[30] Doedel J, Fairgrieve TF, Sandstede B, Champneys AR, Kuznetsov AY, Wang X. AUTO-07P: continuation and bifurcation software for ordinary differential equations. 2007.

[31] Ermentrout B (2002). Simulating, analyzing, and animating dynamical systems: a guide to XPPAUT for researchers and students.

[32] Li W-C, Cooke T, Sautois B, Soffe SR, Borisyuk R, Roberts A (2007). Axon and dendrite geography predict the specificity of synaptic connections in a functioning spinal cord network. Neural Dev.

[33] Borisyuk R, Kalam al Azad A, Conte D, Roberts A, Soffe S (2014). A developmental approach to predicting neuronal connectivity from small biological datasets: a gradient-based neuron growth model. PLoS ONE.

[34] Roberts A, Conte D, Hull M, Merrison-Hort R, Kalam al Azad A, Bhul E, Borisyuk R, Soffe S (2014). Can simple rules control development of a pioneer vertebrate neuronal network generating behaviour?. J Neurosci.

[35] Hull MJ, Soffe SR, Willshaw DJ, Roberts A (2015). Modelling the effects of electrical coupling between unmyelinated axons of brainstem neurons controlling rhythmic activity. PLoS Comput Biol.

[36] Ferrario A, Merrison-Hort R, Soffe SR, Borisyuk R (2018). Structural and functional properties of a probabilistic model of neuronal connectivity in a simple locomotor network. eLife.

[37] Angstadt JD, Grassmann JL, Theriault KM, Levasseur SM (2005). Mechanisms of postinhibitory rebound and its modulation by serotonin in excitatory swim motor neurons of the medicinal leech. J Comp Physiol, A Sens Neural Behav Physiol.

[38] Destexhe A, Mainen ZF, Sejnowski TJ (1998). Kinetic models of synaptic transmission. Methods Neur Model.

[39] Sautois B, Soffe S, Li WC, Roberts A (2007). Role of type-specific neuron properties in a spinal cord motor network. J Comput Neurosci.

[40] Roberts A, Kahn JA, Soffe SR, Clarke JDW (1981). Neural control of swimming in a vertebrate. Science.

[41] Buhl E, Roberts A, Soffe SR (2012). The role of a trigeminal sensory nucleus in the initiation of locomotion. J Physiol.

[42] Boothby KM, Roberts A (1995). Effects of site of tactile stimulation on the escape swimming responses of hatchling Xenopus laevis embryos. J Zool.

[43] Davis A, Merrison-Hort R, Soffe SR, Borisyuk R (2017). Studying the role of axon fasciculation during development in a computational model of the Xenopus tadpole spinal cord. Sci Rep.

[44] Soffe SR, Roberts A (1982). Activity of myotomal motoneurons during fictive swimming in frog embryos. J Neurophysiol.

[45] Li WC, Moult PR (2012). The control of locomotor frequency by excitation and inhibition. J Neurosci.

[46] Kuznetsov YA, Meijer HG, Van Veen L (2004). The fold-flip bifurcation. Int J Bifurc Chaos.

[47] Wang XJ, Rinzel J (1992). Alternating and synchronous rhythms in reciprocally inhibitory model neurons. Neural Comput.

[48] Li W-C, Roberts A, Soffe RS (2010). Specific brainstem neurons switch each other into pacemaker mode to drive movement by activating NMDA receptors. J Neurosci.

[49] Wojcik J, Schwabedal J, Clewley R, Shilnikov AL (2014). Key bifurcations of bursting polyrhythms in 3-cell central pattern generators. PLoS ONE.

[50] Cymbalyuk GS, Gaudry Q, Masino MA, Calabrese RL (2002). Bursting in leech heart interneurons: cell-autonomous and network-based mechanisms. J Neurosci.

[51] Lodi M, Shilnikov A, Storace M (2017). CEPAGE: a toolbox for central pattern generator analysis. Proc IEEE int symp circuits (ISCAS).

[52] Danner SM, Wilshin SD, Shevtsova NA, Rybak IA (2016). Central control of interlimb coordination and speed-dependent gait expression in quadrupeds. J Physiol.

[53] Izhikevich EM (2007). Dynamical systems in neuroscience.

[54] Kepler TB, Abbott LF, Marder E (1992). Reduction of conductance-based neuron models. Biol Cybern.

[55] Ashwin P, Coombes S, Nicks R (2016). Mathematical frameworks for oscillatory network dynamics in neuroscience. J Math Neurosci.

[56] Govaerts W, Sautois B (2006). Computation of the phase response curve: a direct numerical approach. Neural Comput.

[57] Rubin JE, Shevtsova NA, Ermentrout GB, Smith JC, Rybak IA (2009). Multiple rhythmic states in a model of the respiratory central pattern generator. J Neurophysiol.

[58] Lodi M, Shilnikov S, Storace M (2017). Design of synthetic central pattern generators producing desired quadruped gaits. IEEE transactions on circuits and systems I: regular papers.

