# Supplementary material for: Bifurcations of Limit Cycles in a Reduced Model of the Xenopus Tadpole Central Pattern Generator
Source: J Math Neurosci. 2018 Jul 18;8:10. doi: 10.1186/s13408-018-0065-9 (PMC6051957; doi:10.1186/s13408-018-0065-9)
Supplement: Supplementary file 1 — Supplementary material (DOCX 89 kB) [file 13408_2018_65_MOESM1_ESM.docx]

Supplementary material

| dIN/cIN | Rate Function | A ($ms^{-1}$) | B ($ms^{-1}mV^{-1}$) | C (-) | D ($mV$) | E ($mV$) |
| --- | --- | --- | --- | --- | --- | --- |
| Ca | $\alpha_{r}$ | $4/-$ | $0/-$ | $1/-$ | $-15.3/-$ | $-13.6/-$ |
|  | $\beta_{r}(v<-25mV)$ | $1.2/-$ | $0/-$ | $1/-$ | $10.6/-$ | $1/-$ |
|  | $\beta_{r}(v>-25mV)$ | $1.3/-$ | $0/-$ | $1/-$ | $5.4/-$ | $12.1/-$ |
| K-fast | $\alpha_{f}$ | $5.1/3.1$ | $0.1/0$ | $5.1/1$ | $-18.4/-27.5$ | $-25.4/-9.3$ |
|  | $\beta_{f}$ | $0.5/0.4$ | $0/0$ | $0/1$ | $28.7/9$ | $34.6/16.2$ |
| K-slow | $\alpha_{s}$ | $0.5/0.2$ | $8.2e-3/0$ | $4.6/1$ | $-4.2/-3$ | $-12/-7.7$ |
|  | $\beta_{s}$ | $0.1/0.05$ | $-1.3e-3/0$ | $1.6/1$ | $2.1e5/-14.1$ | $3.3e5/6.1$ |
| Na | $\alpha_{m}$ | $8.7/13.3$ | $0/0$ | $1/0.5$ | $-1/-5.$ | $12.6/-12.6$ |
|  | $\beta_{m}$ | $3.8/5.7$ | $0/0$ | $1/1$ | $9/5$ | $9.7/9.7$ |
|  | $\alpha_{h}$ | $0.1/0.04$ | $0/0$ | $0/0$ | $38.9/28.8$ | $26/26$ |
|  | $\beta_{h}$ | $4.1/2$ | $0/0$ | $1/1e-3$ | $-5.1/-9.1$ | $-10.2/-10.2$ |

Table S1. Parameters defining the rate functions of the model neurons rounded to the first decimal digit ($-$ sign that the cell type has no contribution of the specific channel variable).


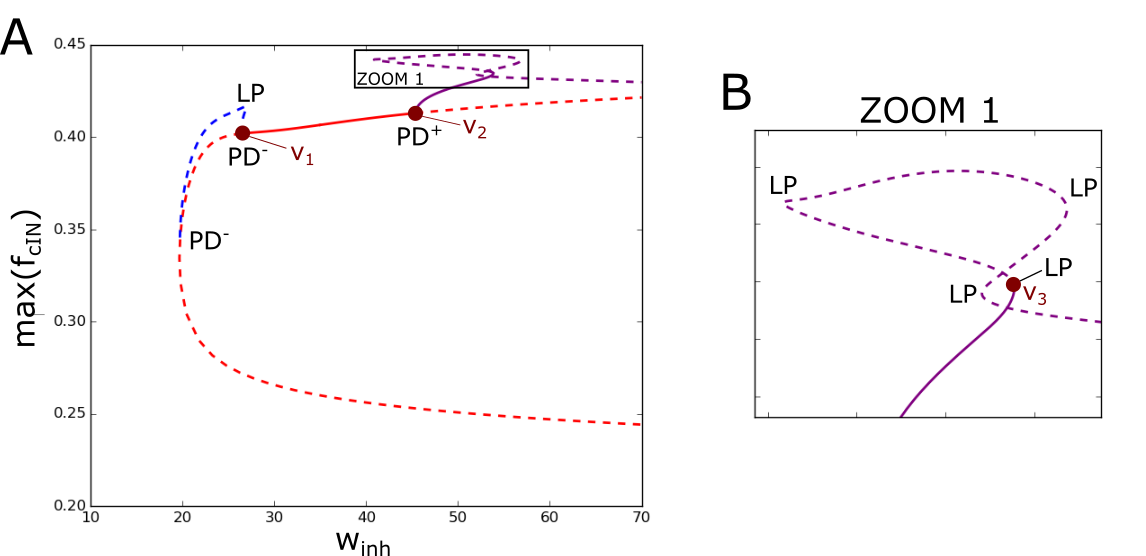


Figure S2. A. One dimensional bifurcation diagram for the SyC (red line) at varying $w_{inh}$ and fixed parameters $w_{ampa}=12nS$ and $w_{nmda}=14nS$. B. Zoom of a selected region to clarify the trend of overlapping bifurcation lines. The SyC is stable for $w_{inh}\in(w_{1},w_{2})$. The SyC loses stability at $w_{inh}=w_{1}$ a PD^-^, and at $w_{inh}=w_{2}$ via PD^+^. For $w_{inh}\geq w_{2}$ the stable 2-SyC originates and remains stable for $w_{inh}\in(w_{2},w_{3})$. At $w_{inh}=w_{3}$ the 2-SyC disappears via LP bifurcation, as shown in Figure 7. All the remaining bifurcation points are the same bifurcations as the ones shown Figure 3 and Figure 5. For a complete description of these points, see Sections 4.3 and 4.4 and Figure 3.
